# A new conception and subsequent taxonomy of clinical psychological problems

**DOI:** 10.1186/s40359-019-0318-8

**Published:** 2019-07-10

**Authors:** Gary M. Bakker

**Affiliations:** School of Medicine, University of Tasmania, Locked Bag 1377, Launceston, Tasmania 7250 Australia

**Keywords:** Case formulation, Clinical psychological problems, Functional analysis, Mental disorders, Symptom networks, Problem-maintaining circles, Taxonomy, Transdiagnostic

## Abstract

**Background:**

A taxonomy of the objects of study, theory, assessment, and intervention is critical to the development of all clinical sciences. Clinical psychology has been conceptually and administratively dominated by the taxonomy of an adjacent discipline – psychiatry’s *Diagnostic and statistical manual of mental disorders* (DSM). Many have called for a ‘paradigm shift’ away from a medical nosology of diseases toward clinical psychology’s own taxonomy of clinical psychological problems (CPPs), without being able to specify *what* is to be listed and classified.

**Main text:**

An examination of DSM’s problems for clinical psychology, especially its lack of clinical utility, and a search for the essence of CPPs in what clinical psychologists actually do, leads to the proposal that: The critical psychological-level phenomenon underlying CPPs is the occurrence of ‘problem-maintaining circles’ (PMCs) of causally related cognitions, emotions, behaviours, and/or stimuli. This concept provides an empirically-derived, theory-based, treatment-relevant, categorical, essentialist, parsimonious, and nonstigmatizing definition of CPPs. It distinguishes psychological problems in which PMCs have not (yet?) formed, and which may respond to ‘counseling’, *clinical* psychological problems in which active PMCs require clinical intervention, and psychopathological problems which are unlikely to be ‘cured’ by PMC-breaking alone.

**Conclusion:**

A subsequent classification and coding system of PMCs is proposed, and expected benefits to research, communication, and the quality of case formulation in clinical psychology are described, reliant upon a development effort of some meaningful fraction of that which has been devoted to the DSM.

## The need for a new conception of clinical psychological problems

The focus, advancement, and direction of any scientific discipline is critically and essentially dependent upon the basic conceptualization that it holds of its subject matter [1–3], and upon the subsequent taxonomy or listing which it develops of that subject matter [4, 5]. Chemistry has its periodic table. Zoology and botany list and categorize species. Astronomy has developed its star charts, galaxy taxonomy, and so forth.

In order to avoid unwarranted assumptions, the objects of systematic observation and experiment, theory development, assessment, and intervention within the science and practice of clinical psychology – it being a *remedial* discipline – may most simply be called clinical psychological problems (CPPs). CPPs have, over time, variously been conceived as instances of demonic possession [6], moral failings [7], deeply-rooted psychodynamic pathologies, observable patterns of maladaptive behaviour, or internal states fundamentally grounded in biology [8]. Each alternative conceptualisation has entailed its own ad hoc or systematised taxonomy of psychological-level problems.

Since the publication of the third edition of the American Psychiatric Association’s *Diagnostic and statistical manual of mental disorders* (DSM) [9] – the first edition to be based heavily on a medical model of ‘mental disorders’ (closely paralleled by the WHO’s *International Classification of Diseases*) – the conception of CPPs as biologically-based internal states has come to dominate, and the DSM has become clinical psychology’s de facto problem taxonomy. This has led to and cemented the assumption that CPPs are solely and entirely mental disorders. This assumption has resulted in many widely recognised and irreconcilable problems for the discipline and profession of clinical psychology.

(The soon to be implemented latest iteration of WHO’s ICD classification – ICD-11 – acknowledges these problems more than DSM-5 does, and its response will be described shortly.)

The publication of the latest edition of this taxonomy, DSM-5 [10], has solved none of the problems alluded to [11–16], prompting the British Psychological Society’s Division of Clinical Psychology to release a consensus statement on psychiatric diagnosis – *Position Statement on the Classification of Behaviour and Experience in Relation to Functional Psychiatric Diagnoses: Time for a Paradigm Shift* [17] – which summarized DSM’s limitations and deficiencies for clinical psychology. It concluded that “the current classification system as outlined in DSM…has significant conceptual and empirical limitations, consequently there is a need for a paradigm shift in relation to the experiences that these diagnoses refer to, towards a conceptual system which is no longer based on a ‘disease’ model” (p.1).

However, beyond suggesting that “such an approach would need to be multifactorial, to contextualise distress and behaviour, and to acknowledge the complexity of the interactions involved” (p.3), and that it should be “in keeping with the core principles of formulation in clinical psychology” [17], the Division did not propose a precise focus or the content of such an alternative conceptual taxonomy that would satisfy this paradigm shift. It is insufficient to point up the limitations of a conceptual model of CPPs if a superior one cannot be proffered, and “at present there is no consensus on what an alternative, universal theory of what maintains and exacerbates psychological distress might look like” [44].

If we are to develop such a radical reconceptualization of CPPs, to foster “a true ‘Kuhnian’ revolution” ([18] p.1935) in clinical psychology, and to develop a subsequent taxonomy of such, then the nature and essence of these new CPPs may be discernible in two ways: (1) The precise nature of the recognised inadequacies and incompatibilities of the mental disorder model for clinical psychology can point us toward a more useful and relevant conceptualization of CPPs; And (2) what clinical psychologists *actually address* in their research and practice may be drawn and distilled to extract the true essence of CPPs.

So firstly, what can the problems and incompatibilities for clinical psychology of psychiatry’s DSM and its ‘mental disorders’ model teach us about the essence of CPPs?

### Psychiatry is more biological-level; clinical psychology is more psychological-level

Psychiatry, as a branch of medicine, has a much greater focus on biological-level assessment, explanation, and intervention than has clinical psychology. Hence, its DSM lists.

‘mental disorders’, ‘diagnosed’ by the identification of a ‘syndrome’ of ‘symptoms’, which are assumed to be manifestations of a ‘pathological condition’ [19, 20].

This ‘nosology of diseases’ [21] based not on empirical evidence, but on clinical authority and historical tradition [22], has been problematic for psychiatry itself [13, 23–26], let alone for clinical psychology. It has been plagued by such major problems as excessive rates of comorbidity [27, 28], which may be an indicator of arbitrary boundaries between its disorders [29, 30], by the broad heterogeneity within its diagnosed groups [21, 31–33], and by the fact that none of the putative underlying disease processes have been uncovered in the 35 years of research since DSM-III was published [25, 34–37]. The search for biological etiology has greatly disappointed [38, 39], suggesting that psychiatric diagnosis has *over*simplified psychopathology [40].

DSM and the ICD, meantime, have been poor guides to even psychopharmacological treatment selection [41, 42], let alone to psychological therapy selection. Psychiatric drugs are frequently prescribed “outside their license”, as when chlorpromazine is administered for anxiety or insomnia, thioridazine (another antipsychotic) for alcohol withdrawal, and benzodiazepines for “pretty much everything…The classification of mental health conditions gives us a false sense of order…It has little or no relevance to psychotropic drug action” ([43] p.225).

As part of the development of ICD-11, First et al. [44] surveyed 1764 mental health professionals, mainly psychiatrists, and found that the majority used ICD-10 or DSM-5 for administrative or billing purposes only. They rated such taxonomies as least useful for treatment selection and determining prognosis.

Psychiatry’s own response to these acknowledged problems has been to redouble its conceptual biological insistence. For example, the National Institutes of Mental Health are developing a Research Domain Criteria (RDoC) framework [45] which, even more than DSM, conceptualises mental illnesses as brain disorders (latent disease constructs), but which seeks to identify them through objective behavioural tests and neurobiological measures such as genetic tests and neuroimaging, rather than through topographical symptom checklists. This response to DSM’s failures has been described as a shift from the biopsychosocial model of mental disorders to a “bio-bio-bio model” [46]. It is much more a framework for biologically-oriented research [47] than a clinical replacement for the ICD or DSM [48]. While it tries to be more etiological and dimensional than those systems, its clinical usefulness lies well into the future [49].

The RDoC approach is therefore not at all a solution to clinical psychology’s problems with the conception of CPPs as diagnosable mental disorders, which are less to do with the technical limitations of diagnosis and more to do with a conceptual mismatch. The proposers and developers of the RDoC project have attempted to circumvent the problem that heterogeneous symptom profiles among diagnosed groups are likely to encompass a large number of biologically distinct entities [50]. But clinical psychologists’ concerns are that these groups are likely to encompass a large number of *psychologically* distinct entities.

So even were the RDoC project to improve diagnostic reliability, validity, and clinical utility for psychiatrists, it would still offer no greater attraction to clinical psychology. Clinical psychologists as a group are not as biologically focused or trained, do not prescribe medications or administer ECT, and in fact in practice rarely and reluctantly diagnose [51–53]. Instead, they construct *case formulations* at a *psychological* level [54].

The idea that CPPs can and should be reducible to presumed underlying neurobiological conditions which are somehow more basic, real, or ‘scientific’ than psychological-level formulations is not helpful [12], not logical [55], and, for almost all CPPs, is theoretically premature [56]. The psychological and the biological are *different levels* of analysis, assessment, and intervention [57], and any alignment of phenomena at these two levels is, by definition, correlational, not causal [55]. It is no more likely that all CPPs will be reduced in the future to neurobiological conditions than that the geological study of earthquakes will be reduced to molecular theory ([57] p. 508).

While it is possible and desirable to theoretically unify the social sciences and biology, the notion of abandoning the principles, theories, vocabulary, and laws of the social sciences in favour of lower-level terms is a “preposterous” proposition [58], which would result in such theses as “A Comparison of Keats and Shelley from the Molecular Point of View” or “The Role of Oxygen Atoms in Supply-Side Economics”. Such “greedy reductionism” can arise when “in their zeal to explain too much too fast, scientists and philosophers often underestimate the complexities, trying to skip whole layers or levels of theory” ([58] p.82). “Mental disorders may be studied at different levels of analysis (e.g. molecular genetics, neurochemistry, cognitive neuroscience, personality, environment), and no level is inherently superior or fundamental to any other” ([12] p.856).

Clinical psychologists, when they operate within an adjacent level of analysis – in this case a psychiatric one – will lose a large, perhaps critical, amount of *psychologically*-relevant information. “Psychiatrists using the DSM diagnosis ‘major depression’ tend to mingle bereaved patients with both those afflicted by classic melancholia and those demoralized by circumstances” ([59] p.1854). So when clinical psychologists allow themselves to be diverted from the study and psychological-level formulation of CPPs to research into the treatment of DSM-diagnosed mental disorders, this means that a 19 year old survivor of 14 years of sexual abuse within her dysfunctional family, who is now sad and amotivational every day, will be regarded as experiencing precisely the same CPP as a 73 year old recently bereaved widower who is also sad and amotivational every day, because these people share some ‘symptoms’ – some topographical similarities. They will also find themselves in the same experimental or control group in a clinical trial of a particular cognitive therapy or antidepressant medication, and conclusions about efficacy will then be extended to other people with even more diverse CPPs, because they allegedly have the same mental disorder.

It is highly likely that some CPPs currently regarded or labelled as mental disorders are most usefully assessed, diagnosed, and treated within a medical model, but that some do not conform well to this level of analysis, and will respond better when assessed and addressed at a psychological level [59–61]. “Psychiatric diagnoses differ in the sorts of categories that best capture them” ([60] p.204). Some may be more categorical than others [62]. There is some evidence, for example, that anorexia nervosa may be much less culture-bound and more heritable than bulimia nervosa [63], and so may be less socially constructed, more categorical, and a different ‘kind’ of thing. Schizophrenia and a simple reactive dog phobia are also likely to represent different classes of CPP in this light. The former more comfortably rests within a taxonomy of ‘mental disorders’ such as the DSM. A reactive dog phobia, on the other hand, may be more conceptually concordant with clinical psychology’s own parallel purely psychological-level taxonomy of CPPs.

It will be a long time – if ever – before a complicated bereavement is fully explained by reference to a particular neural bundle, or treated solely with a localized electrical zap or a ‘complicated bereavement pill’. Clinical psychology and biological psychiatry are different disciplines, operating at adjacent but different levels of analysis, and neither should subsume the other.

Our new conception of CPPs, and its subsequent taxonomy, will therefore centre on *psychological-level* states and processes – involving cognitions, emotions, behaviours, and situations or stimuli – and not on biological-level ones.

### Mental disorders are social constructions; they have no essence

Another major problem with equating CPPs with mental disorders is that this subsumption represents relegation to a less developed, less theoretically robust, less therapeutically relevant level of analysis. This is an inevitable consequence of the fact that, whereas our theoretical knowledge of the processes, functions, and mechanisms underlying CPPs has grown greatly, DSM’s listing of mental disorders began and has remained stolidly atheoretical [8, 32, 64, 65]. DSM has made no claims about underlying mechanisms, functional processes, pathophysiology, etiology, and hence treatment implications of its mental disorders, and is therefore a “weak medical model” [66].

This deliberate policy was originally so as to accommodate a large number of theoretical orientations from a range of professions or disciplines [32, 35], but also more recently because, as previously described, the medical model has largely failed to further our understanding of the heterogeneous assortment of disorders the DSM lists [21]. The sluggish pace of discovery in psychiatry has been attributed, in part, to the limited validity and the arbitrariness of traditional diagnoses [67].

So, whereas a clinical psychologist will see a CPP involving problematic social anxiety, for example, as a psychological-level persisting negative process that requires case formulation and specific subsequent psychological-level intervention, according to DSM a Social Anxiety Disorder is a state or condition identified (but not explained) by its symptoms. How do we know that Bill has a Social Anxiety Disorder? He shows enough symptoms. What caused these symptoms? His Social Anxiety Disorder. There is no evidence that the mental disorder or mental illness called “Social Anxiety Disorder (Social Phobia)” (DSM300.23) actually exists. It has no ‘essence’. There are no reliable or validated biological markers or measures outside clinical psychological judgement that can detect this illness. It is defined by its effects, which are attributed to its existence [52]. It is an assumption – a convention – constructed for sociological or political reasons, just as the disease model of alcohol problems and the chemical imbalance theory of depression were. They were developed in an attempt to reduce stigma or encourage people to take their antidepressants. But no evidence supportive of their veracity has emerged since.

So the concept of ‘mental disorders’ is inadequate to supplant that of CPPs because it is descriptive only – not at all explanatory. But such disorders are therefore also inevitably vaguely and arbitrarily defined and demarcated. “Diagnostic criteria [in psychiatry] shift and sway like in no other area of medicine” [43]. The DSM meanders between at least seven different criteria in distinguishing non-problems from problems-deserving-therapy (mental disorders). At different times DSM specifies: (i) A certain symptom cluster. Three or more symptoms from a field of seven are required to diagnose an Antisocial Personality Disorder. (ii) A certain level of distress. In OCD “the obsessions or compulsions… cause clinically significant distress”. (iii) A level of dysfunction. This is required for a diagnosis of Specific Phobia. (iv) A certain type of etiology. This marks a Posttraumatic Stress Disorder (PTSD) or a Substance-Induced Sexual Dysfunction. (v) A statistical deviation. To diagnose a Female Orgasmic Disorder requires a “marked infrequency” of orgasms. (vi) The chemistry involved. This determines an Alcohol-Related Disorder. And (vii) duration is a criterion determining the presence of an Acute Stress Disorder or Dysthymia [10]. One or two clear conceptual criteria to distinguish CPPs from ‘normal problems in life’ would be much preferred.

When arbitrary categories are forced onto dimensional phenomena like symptoms, then both reliability and validity have been shown to suffer [68–70]. Not only have no biological markers for the common mental disorders been uncovered, but this arbitrarily interchangeable collection of criteria for determining their presence means that not a single mental disorder has been established as a discrete categorical entity, as opposed to a dimensional outlier [71, 72].

This conceptual vacuum has left ‘mental disorders’ as merely social constructions [60], ‘open concepts’ [73], or ‘practical kinds’ [15, 40] without a true defining essence [27], and has resulted in intractable and interminable debates among psychologists and psychiatrists as to what ultimately defines a mental disorder [53]. Hence we have seen the inclusion and then exclusion in 1974 of homosexuality [74], and historical arguments over ‘childhood masturbation disorder’ and ‘lack of vaginal orgasm’ [75]. Right up to today, clinicians and researchers have criticized the seemingly unsystematic and arbitrary addition in DSM-5 of “bizarre new illnesses” such as Excoriation Disorder (skin picking), Major Depressive Disorder 2 weeks after a bereavement, or Somatic Symptom Disorder when one is adjudged ‘too upset’ when told of a cancer diagnosis [14]. New diagnoses have mostly arisen when a few influential insiders have decided that a new category would be clinically meaningful and handy, and have lobbied for its inclusion [76].

Unlike mental disorders, CPPs must be ‘natural kinds’ of things in the world [77, 78] with a defined essence [79], that delineates a *category* else they revert, like mental disorders, to being merely *dimensional* variants of normality [80, 81]. Such a capitulation, in which we merely choose to call something a mental disorder or a CPP when it has gone far enough to bother us, is a surrender to the opposite, postmodern extreme [82, 83] in which problems may be explained by a “medical narrative” no more or less legitimately than by a learning theory-based narrative, or by “socio-political, spiritual or paranormal explanations” [14]. When a conceptual over-reach leads to the abandonment of the scientific method altogether, the need for a ‘paradigm shift’ is urgently indicated.

It is generally recognized in all fields of scientific endeavour that it is preferable that concepts be essentialist rather than undefined or arbitrary [61], and categorical rather than dimensional [84] or merely nominal [81]. For example, it is epistemologically and clinically much more useful if disorders can be conceptualized in terms of pathological processes rather than being solely descriptive [81]. After a century of successful, progressive research and practice in clinical psychology, we are more than confident that CPPs exist in the real world [8], and are qualitatively different from normality, but depend on a client’s, a therapist’s, or society’s evaluation. That is, that CPPs are a form of ‘harmful dysfunction’ [75] where the harm is a judgement, but the dysfunction is objective [85].

Though CPPs are almost universally and intuitively regarded as qualitatively different from normality, much theoretically important research has focussed on *dimensional* constructs and their relationships with (even barely valid) categories such as mental disorders. This has occurred because each approach (dimensional or categorical) has its advantages in different contexts [86]. When testing hypotheses, dimensional measures in research retain more information than categories, especially for phenomena that are distributed fairly continuously, and with unclear boundaries [70].

Categories are also highly dependent on appropriate cutpoints. We know that cutpoints for most mental disorders are fuzzy and somewhat arbitrary. This is why they are barely categorical. And this is also why most dimensional models of psychopathology focus on the personality disorders [87]. They have even more ‘fuzzy boundaries’ than the other mental disorders.

Hence, recognising the problems that DSM-5 and ICD-10 have had with arbitrary thresholds, large category overlaps, and low clinical utility, the new ICD-11 has adopted a dimensional approach to personality disorder classification with 5 trait qualifiers: Negative Affectivity, Detachment, Dissociality, Disinhibition, and Anankastia [88]. Whereas DSM-5 and ICD-10 require a quota of criteria to be met (e.g. 5 of 9) to define a disorder, ICD-11 diagnoses Personality Disorder via global evaluation of personality functioning, where the clinician may specify 5 evidence-based trait dimensions that contribute to the unique expression of personality disturbance.

It has been claimed that WHO’s ICD-11 is less entirely based on tradition and authority than the DSM [48] as it is more empirically derived, in that dimensional approaches correspond better to the observed data than do purely categorical ones [47]. Continuous (dimensional) measures of psychopathology have been found to increase both the reliability and the validity of assessments over discrete (categorical) measures [70]. Subsequent to this, Reed, Sharan et al. [89] found the reliability of ICD-11’s guidelines to be superior to that previously reported for equivalent ICD-10 guidelines, and Reed, Keeley, et al. [90] found clinicians rated the clinical utility of ICD-11’s diagnostic guidelines very positively with regard to ease of use, but still poorly for treatment selection.

Note that the dimensions of personality *functions* here refer to *processes* rather than static traits or features, and therefore this assessment of *functioning* more closely approximates a functional analysis than a diagnostic checklist. And it therefore can potentially more closely lead to therapeutic conclusions [88]. “[T]reatment should target what the Personality Disorder *does* to the patient (i.e., severity), as we cannot change what it is (i.e., traits).”

These changes of direction embodied in the soon to be implemented ICD-11 are a response to the recognition that psychotherapeutic interventions are often transdiagnostic [91]. However, they offer only a clinically useful dimensional overlay to a categorical ‘mental disorder’ conceptualisation – not a paradigm shift. “Advances in psychiatric research in general, and progress in nosological science in particular, will continue to be iterative….but no evidence has suggested that genetic or other biological information will lead to a paradigm shift in diagnostic classification in the immediate future” ([91] p.7).

However, clinicians and clinical researchers generally require a categorical approach [86] because they need to constantly decide whether to treat or not, whether to hospitalise or not, or which drug or psychotherapy to apply, or not. ICD-11, as well as DSM-5 must be predominantly categorical for administrative and treatment selection purposes. Such decisions are categorical, even if the data underlying them is dimensional [48].

For our purposes, the cutpoints and criteria for the determination of the presence of a CPP must be clearer, better validated, and more essentialist than those for the diagnosis of mental disorders. Dimensional measures give us indications as to the *statistical* significance of a relationship or an outcome, but categorical approaches tell us more about their *clinical* significance – whether a *qualitative* difference has been created or detected, or merely a possibly-trivial change in one psychometric score.

The usual structure of research projects in this field has been to explore the relationships between dimensional constructs and factors, such as ‘anxiety sensitivity’, and categorical problems, such as generalised anxiety disorder (GAD) [92] even though GAD as a construct has poor validity, dubious reliability, and an arbitrary cutpoint. Hence, it has been noted that the cycle of progress that is meant to occur between dimensional research in psychiatry and clinical diagnostic accuracy has not eventuated [86, 93].

When dimensional measures are used in research on mental disorders, the richer data has provided even more evidence that DSM diagnoses correlate poorly with these measures, and that they better predict certain psychological *processes* than psychopathological conditions. For example, Melville et al. [94] found that problem behaviours, rated or scored dimensionally, among adults with intellectual disabilities, loaded in a factor analysis within an emotion dysregulation/problem behaviour dimension, not within depressive, anxiety, organic, or psychosis dimensions or factors.

Similarly, when the relationship between the dimensional construct ‘dispositional negativity’ and adverse outcomes such as ‘emotional disorders’ is studied [95], a “dynamic cascade of processes” – presumably amenable to functional analysis – is uncovered; not a simple relationship with one or two diagnosed mental disorders. Further, when dimensional psychopathology measures are factor analysed across a population, a strong *general* psychopathology factor emerges. Carragher et al. [96] concluded from this that *transdiagnostic* treatment approaches are indicated and warranted, and the domain of psychopathology should be restructured in an empirically-based manner, as has recently commenced through the HiTOP consortium [69].

So dimensional approaches to CPPs or to mental disorders can be very valuable, especially in research. But it would be preferable that any psychological-level alternative to the categorical diagnosis of mental disorders be a more evidence-based and essentialist *categorical* conception of CPPs.

Therefore, any new conception of CPPs must, by contrast with DSM’s mental disorders, be theory-rich, evidence-based, problematic-by-judgement, real-by-nature (essentialist), categorical (qualitatively distinct from normality) according to only one or two reliable and valid criteria, and must recognise various conceptual kinds of psychological problem.

### Treatment-relevant case formulation versus nominative diagnosis

With further regard to the DSM system’s natural taxonomic inadequacies for clinical psychologists, because the vast majority of psychologists seek to intervene at a psychological level (i.e. in situations, thoughts, feelings, and behaviours), they are much more interested in developing a process- or functional- or mechanism-focused case formulation than a symptom-derived diagnosis. Clinical practice is predominantly theory-based rather than manual-prescriptive [53].

Whereas psychiatric practice is more and more dominated by the process of deriving DSM diagnoses, [32, 97], an examination of the UK’s *Generic Professional Practice Guidelines* for psychologists, or the APS’s *College of Clinical Psychologists Course Approval Guidelines,* shows that “training programs for clinical psychologists emphasize formulation rather than diagnosis” ([52] (p.448). Formulation is fundamental to clinical psychology in the same way that diagnosis is fundamental to psychiatry [51], and DSM diagnosis is often irrelevant to psychological practice [20, 54].

Among the advantages of the case formulation approach identified by the BPS’s Division of Clinical Psychology [17, 98] are much greater treatment-relevance, strengthening of the therapeutic alliance, normalization of problems, providing a sense of hope, reducing blame, and increasing collaboration and empathy.

With regard to treatment-relevance and clinical utility, even for psychiatrists the DSM “describes a collection of disorders, not an integrated system of psychopathology” ([31] (p.147). Many existing diagnoses encompass multiple pathological processes [33]. DSM’s search for reliability of diagnosis at the cost of theoretical integration and validity [84] plus its high rates of comorbidity, high frequency of “Other Specified/Unspecified” (previously “Not Otherwise Specified”) diagnoses, and divergent and overlapping criteria sets make for little guidance in choice of treatments [25].

But especially for clinical psychologists, who are more concerned with psychological-level case formulation, in most cases a DSM diagnosis tells one little about etiology, course, choice of treatment, or treatment response [52, 99–103]. “Identifying a disorder by its symptoms does not translate into understanding it. Clinicians need some heuristic concept of its nature, grasped in terms of cause or mechanism” ([104] p.1845).

A clinical psychologist basing treatment on a DSM diagnosis in place of a case formulation is like a dietician assessing the adequacy of a person’s diet by taking a height measurement, instead of interviewing the person. Height measurement is a much more reliable, consistent, brief, and precise process, but far too much validity and treatment relevance are lost.

Therefore, the new conception and taxonomy of CPPs must be formulation-relevant and treatment-relevant, and not just a listing of surface-symptom-defined diagnoses.

### Processes or mechanisms versus topographical description

Tucker [97] admitted that, by contrast with the rest of modern medicine, in psychiatry “we are still doing pattern recognition” (p.159). This approach falls down when the same pattern or topography can be established by diverse processes, or when different topographies can come from the same process [35]. These phenomena have come to be known as the problems of multifinality and divergent trajectories [105]. Multifinality [106] refers to how one general transdiagnostic risk factor or process can result in several different disorders, as when stress [107] can contribute to the development of several different CPPs involving anxiety, depression, or alcohol abuse. Divergent trajectories occur when more proximal or moderating variables, such as attentional biases, result in OCD in one person, but a sleep disorder in another [105].

The DSM system has ignored these issues and focused on final symptoms and their topography, saying nothing about mechanisms. We need to comprehend psychopathological disorders “not simply by their outward show but by the causal processes and generative mechanisms known to provoke them” ([104] p.1855).

Major problems such as treatment irrelevance and excessive unexplained comorbidities have resulted [28–30]. In the clinical psychological literature these comorbidities, such as between Major Depressive Disorder, Generalized Anxiety Disorder, and Dysthymia [108], are assumed to reflect the many similarities of inputs, symptoms, and processes among the various models of particular CPPs.

All of these factors have led to the recent development of transdiagnostic models of psychopathology [109–111] which seek to identify fundamental processes underlying multiple, often comorbid, psychopathologies [105, 112].

Mechanisms across the disorders, such as negative attentional bias [113], experiential avoidance [114], safety behaviours, or rumination [115] have been studied, and transdiagnostic treatment programs that target these processes rather than individual diagnoses have then been developed [4, 109, 116, 117]. This naturally follows the finding that more than half of patients who present with depressive disorders also have elevated comorbid anxiety symptoms, and that, when psychotherapy for depression is undertaken, anxiety can be significantly ameliorated [118]. Very few studies have examined this issue because the literature is strictly structured around individual psychiatric disorders [118].

Current transdiagnostic approaches circumvent the problem of the plethora of manualized treatment programs for a growing number of specific diagnoses [119], so that the training of therapists and development of treatment packages can be more parsimonious [4].

The taxonomic arm of this empirically-based transdiagnostic movement – the Hierarchical Taxonomy Of Psychopathology (HiTOP) consortium – grew out of the psychological study of individual differences [120]. Its rich vein of studies [121–123] establishing an alternative *dimensional* organization of psychopathology helps to overcome such problems with traditional nosologies as the issue of arbitrary thresholds and subsequent loss of information, ensuing reliability problems, diagnostic heterogeneity, theoretically disruptive high comorbidities [120], and exclusion of undiagnosable ‘subthreshold’ people with serious CPPs [49, 69, 124].

The emergent HiTOP dimensions form a hierarchy with five levels (symptoms, syndromes, subfactors, etc.), and can thus help explain why disorders from different classes respond to the same treatment (e.g. social anxiety responding to antidepressants) [49]. In this way it is a critical part of the transdiagnostic movement.

But the HiTOP hierarchical dimensional models of classification, though guided by research [120] are still the result of a consensus among the consortium [49], require interpretation by human experts [48], and the approach suffers from all the problems of a dimensional taxonomy. It has not, to date, been used clinically, as the consortium has yet to develop meaningful cut-off points for pathology [49]. It can still only offer a dimensional elaboration, based on symptom measurements, on top of a categorical ‘disorder’ model [48], because it still does not implicate proximal causes for, and the ‘essence’ of, CPPs. It is a descriptive phenotypic model, and does not directly incorporate etiology and underlying mechanisms [49]. It shares many of the same constructs with the categorical model frameworks [120] such as a focus on ‘mental disorders’.

For example, when Nolen-Hoeksema and Watkins [105] have suggested ways to explain multifinality and divergent trajectories in terms of distal, proximal, and moderating causes or risk factors (p. 592), they have done so via a flow chart resulting in (DSM-type) ‘Disorder A’, ‘Disorder B’, and ‘Disorder C’. Despite emphasising that we need more focus on the precise *mechanisms* involved (p.591), the transdiagnostic movement still regards an Anxiety Disorder as the same CPP whether it has arisen through a mechanism of avoidance or of rumination. The necessary ‘paradigm shift’ would see these two situations as different CPPs.

As psychological interventions increasingly target mechanisms, such as specific cognitive dysfunctions, rather than symptom-based mental disorders, a new comprehensive conceptual framework to assemble the results of psychotherapy research will be required [125].. The transdiagnostic movement has not to date offered a ‘paradigm shift’; only a useful extra (dimensional) layer, such as allowing for variables such as ‘neuroticism’ or ‘extraversion’ in treatment selection [119], on a categorical ‘mental disorder’ conceptual system.

But also, these transdiagnostic processes and properties are *dimensional* responses to problems with the categorical assumptions of DSM [64]. The assumption is that the heterogeneous disorders in DSM are made up of dysfunctional versions of processes that vary along continua in the general population [45, 126]. For example, attentional bias toward negative information is common in people without [113]. Within the transdiagnostic movement to date, such a bias cannot be regarded as essential or diagnostic. So there remain problems of cut-off points, a quality distinction between different problems rather than a quantity distinction, and the very definition or essence of CPPs. A *categorical* conception of CPPs is preferable [61, 84, 86]. It is much preferable that CPPs, unlike mental disorders, display an essence – that they be more than just ‘worse than normal’.

It has been argued that dimensional data can lead to actionable ‘diagnoses’ in medicine [69], so why not in clinical psychology? For example diagnoses are determined, and treatments initiated, from blood pressure measurements and fasting glucose levels using indicative ranges of scores. However, even in medicine, this is regarded as second best. It is much preferable to uncover some clear, qualitatively distinct pathology such as an infection or a lesion, than to find that a score looks too high or too low. Is it better to treat every adult person under a height of 4′6″ with growth hormone, or to reserve this treatment for people who are not producing their own growth hormone?

Hence, the new conception of CPPs will focus on *mechanisms* and *processes*, not states or conditions. But beyond the current transdiagnostic movement, it will regard the operation of these processes as essential, definitive, and ‘diagnostic’. Thus a categorical conception will emerge, not a merely dimensional one.

### Some CPPs are clearly not mental disorders

In examining what clinical psychologists actually address in research and practice, a stark example of the non-equivalence of CPPs and mental disorders can be found in the fact that clinical psychologists address relationship, marital, and family problems using the exact same assessment and treatment models as for, for example, anxiety or depression problems. Such interpersonal situations clearly cannot be conceptualised as internal mental disorders, and so DSM has relegated “relational pathology” to a terse footnoted ‘V’-code listing, an omission long lamented [127, 128]. As a bizarre and unfortunate consequence, when a clinical psychologist sees a couple or family in the Australian Medicare system they are not eligible for a fee rebate unless one attending party has been given a mental disorder diagnosis by the referring medical practitioner and is being treated for this. ‘No blame’ relationship therapy will not be rebated.

A similarly bizarre and unfortunate result of the conceptual medicalization of CPPs arises with parenting problems. Patterson [129] has described how parent-child interactions frequently directly reinforce deviant behaviour, and he has outlined the role of parent-child discipline practices in the development and maintenance of aggressive behaviour in children. These insights led to the development of the most empirically supported treatment for such problems – Parent Management Training [130]. But, again, to be eligible for a rebate in Australia, not only must the child have a diagnosis of, for example, Conduct Disorder, but the child *must attend* each consultation. The assumption is that the problem resides *within the child*, as would a lesion or infection, and so the mental disorder must be in attendance for treatment to be conferred.

However, perhaps the largest class of CPPs effectively addressed by clinicians, but barely researched because they are not ‘mental disorders’, lies in the third to half of all people who seek clinical psychological help but cannot be given a clear diagnosis because their problems do not fit criteria and categories neatly [131]. They may be ‘subthreshold’ [132], or ‘subclinical’ [133], or situation-specific (such as being evidenced only at work). High levels of distress commonly occur in the absence of a diagnosable condition [134], as when one or two symptoms occur very strongly, but three or more are required for a diagnosis [135]. Should clinical psychologists turn away people presenting with such CPPs because they do not have a diagnosed mental disorder?

Therefore, not all CPPs are internal mental disorders detectable and definable by a certain intensity of symptom presentation. They are more likely to be particular sorts of psychological-level processes, which can occur *between* people as well as within them.

### Social consequences of seeing all CPPs as mental disorders

Promotion of the disease model of CPPs has often occurred in an attempt to ameliorate the serious stigma consequent upon the ‘moral failing’, sinfulness, or demonic possession models of CPPs [136, 137]. The medical model has been advanced as a simple solution to the “brain or blame” dilemma or the “chemistry or character” dichotomy as to whether a person’s mental suffering is real, or they should be told to pull themselves together [14].

Clinicians’ models affect the community’s beliefs and hence sympathetic or stigmatizing attitudes [8]. Psychiatry was aware of this when it promoted the disease model of alcoholism in an attempt to reduce stigma and punitive responses, and increase treatment takeup and compliance [138]. Support groups have used the fact that the concept of mental illness has been arbitrarily defined to agitate for problems such as depression or alcohol dependence to be regarded as diseases, hoping to reduce stigmatization and increase service or research funding [85].

However, in many areas this strategy has backfired, and the personal and social consequences of a psychopathological label have proven to be negative, fatalistic, adverse, and stigmatizing [139]. The “disease like any other” campaigns to convince the public that mental disorders are non-volitional biological illnesses for which sufferers do not deserve blame and discrimination have been “an unequivocal failure in reducing stigma” ([12] p.852). For example, with regard to attitudes to depression and schizophrenia, Schomerus et al. [140] found that (a) belief in the biomedical model has increased, (b) acceptance of medical treatment has increased, but (c) attitudes toward people with mental disorders has *not* improved.

A diagnosis of a mental disorder can often be a cause of disempowerment and social exclusion [141], and may label the person rather than the problem [142]. A diagnosis of mental illness is known to negatively affect self-identity, attract stigma [143], result in a negative prognosis, and engender isolation [144, 145]. People who believe that mental distress is a kind of biological illness are more likely to see psychiatric patients as dangerous and unpredictable [146, 147]. They may blame less, but will fear and avoid patients more [148], and will assume a worse prognosis [147, 149].

Such deleterious consequences are exacerbated by DSM’s assertion that all of the following are examples of the one kind of thing. They are all equally ‘mental disorders’: Mild Tobacco Use Disorder, Schizophrenia, Female Orgasmic Disorder, Delirium, Restless Legs Syndrome, Alzheimer’s Disease, a Spider Phobia, and Opioid Intoxication. Admitting to sadness 2 weeks after one’s spouse has died can put one in the same class, conceptually, as a paranoid schizophrenic, a smoker, a person suffering a panic disorder, or a violent psychopath.

These consequences of problem assessment and problem formulation are not inevitable. It has been claimed that a psychological case formulation or functional analysis approach both avoids the problem of stigmatization [52] and the abdication of responsibility [150] of a mental disorder diagnosis. This provides further reason that the new conception of CPPs needs to be psychological-level and formulation-based.

### Research on CPPs versus mental disorders

Although in practice clinical psychologists formulate much more than they diagnose, almost all research in the discipline ignores this fact. To be considered methodologically sound, and hence to qualify for funding, almost all psychotherapy research must be undertaken with formally diagnosed subjects with the intention of ‘curing’ them of their mental disorders by removing their symptoms. However in real-world clinical practice case formulation guides treatment, which targets psychological processes, not symptom profiles. Treatment outcome measured by “escape from diagnosis” is in this light arbitrary, misleading, and inadequate.

Research trials have typically treated highly selected groups with a single diagnosis, while in clinical practice patients have many comorbidities and atypical symptom profiles [54, 119]. Clinicians are more likely to apply several interventions, and will base this on the individual case formulation they have developed, on the assumption that each technique is targeting something different. When experimental subjects are merely diagnosed and then randomly allocated to comparative treatment groups, they will have an undetermined distribution of relevant underlying mechanisms [151]. A ‘package’ approach ignores basic psychological science and the individual needs of individual clients, is atheoretical, and alienates research from clinical practice [151].

Important comparative studies on various CBTs for depression, such as cognitive therapy (CT) versus behavioural activation (e.g. [152, 153]), or for anxiety problems, such as exposure therapy (ET) versus CT (e.g. [154, 155]), have not been able to find consistent differences between comparative treatments [151]. Michelson et al. [156], for example, were unable to separate the benefits of cognitive, behavioural, and psychophysiological treatments for agoraphobia, though all three were superior to a wait-list control. This is unsurprising, though, when subjects are DSM-diagnosed and then randomly allocated to groups, as though they all have the same CPP. It *assumes* a diagnosis-to-treatment-selection link. This is an example of theory governing the nature of research. However, the medical model of DSM is so entrenched that many researchers would not even see this difference ([21] p.157).

“There are undoubtedly many functionally distinct subtypes of patients currently mixed together in popular diagnostic systems” ([102] p.971). For example, agoraphobics may have a classically conditioned fear of separation, or a fear of panic attacks. Further, this latter fear may in turn be of medical catastrophes or of social embarrassment [151]. Over a quarter century ago, Wolpe [157, 158] warned that such neglect of individual differences in the dysfunctional processes that occur within a diagnostic group puts us in danger of making a mockery out of group treatment outcome research.

A major motivation of the HiTOP consortium has been the fact that randomized controlled trials (RCTs) rarely show superiority among thoughtfully conceived treatment packages [120], and that research has found that many interventions can be beneficial with a host of problems regarded as distinct categorically [118].

This problem has been thoroughly outlined by Smith, McCarthy, and Zapolski [159], who have pointed out that assessing the effect of CT versus ET on a DSM-defined ‘Depression’ group is an example of assessing the relationship of a construct or variable with another multidimensional construct or measure (such as PTSD or Neuroticism) which has *multiple* (diagnostic) criteria. The resultant composite correlation will be an *average* of the correlations with each of the dimensions or criteria, each of which could correlate quite weakly with the others.

The power of RCTs is seriously compromised when the groups that subjects are randomized into are vaguely or spuriously defined. “With heterogeneous treatment effects, the ATE [average treatment effect] is only as good as the study sample from which it was obtained” [160]. This is why researchers have begun to focus on transdiagnostic mechanisms of intervention [161]. “Diagnostic heterogeneity compels the clinician to go beyond the assigned diagnosis and generate individual-level formulations that are not codified in the diagnostic scheme” ([120] p.6).

It will be of much greater benefit when we are able to assemble research results into clinical guidelines not on ‘the treatment of Depression’ or ‘of Bulimia Nervosa’, but on psychological interventions with CPPs A and B, defined by mechanisms, which may cross diagnoses or differ within a diagnosis. For example, we know that targeting specific mediating cognitive processes in a social phobia is more effective than standardized generic cognitive-behavioural treatment [162], because the mental disorder ‘Social Anxiety Disorder’ can encompass a number of (mechanism-defined) CPPs.

### Conclusions

A new conception of CPPs must therefore be: (a) A *psychological*-level one (i.e. involving cognitions, behaviours, emotions, and situations); (b) Psychologically theoretically rich and evidence-based; not a postmodern ‘categories-by-convention-only’ model. It must define an essence. If it comprises a ‘harmful dysfunction’ (Wakefield, 1992), then its harmfulness must be a matter of subjective judgement, but its dysfunction must be defined objectively; And according to only one or two criteria, not a hodgepodge of them; (c) Categorical, rather than merely dimensional; (d) Encompassing of all problems currently appropriately and successfully addressed by clinical psychologists; not merely diagnosed mental disorders; And (e) better at avoiding the stigma and responsibility-confusion problems which have been exacerbated rather than ameliorated by the disease model.

## Seeking the essence of clinical psychological problems

By examining what clinical psychologists actually research and address in their clinical practice, we have come quite close to uncovering the essence of CPPs. Thus far we are clearer about what constitute ‘psychological problems’.

### ‘Psychological problems’

Clinical psychology, like forensic psychology or clinical neuropsychology, is an applied *remedial* discipline. To remedy is to rectify or make good, to cure or heal, to put right or restore, or to counteract or remove. Therefore the new taxonomy will list problems – negative ‘states of affairs’ that are undesired, aversive, inappropriate, maladaptive, or dysfunctional. This loose listing of potential criteria is an indication that the ultimate judgement as to what constitutes a ‘problem’ will inevitably be largely subjective and value-laden, based on ‘presenting problem’ (the client’s standpoint), social norms (society’s standpoint), or psychometric measures (the therapist’s standpoint). Unlike mental disorders, CPPs will not be *of their nature* problematic. They will have to be *deemed* problematic.

CPPs are by definition at a *psychological* level of analysis. That is, at the level of stimuli, cognitions, emotions, and behaviour. Therefore, the new taxonomy to be proposed will not be a listing of biological dysfunctions or of problems faced by communities, cities, nations, or the human species. Sociologists and anthropologists can work at such taxonomies.

It may include, however, problems at an interpersonal, couple, or family level. Clinical psychology has studied these, and does provide remediation at this level. In this respect CPPs are further distinguished from mental disorders, because the biological level of analysis, which DSM’s mental disorders aspire to, is conceptually as well as practically discordant with relationship problems. Very few would recommend that we medicate a faltering relationship.

### ‘Clinical Psychological Problems’

But what makes psychological problems *clinical*? That is, what makes them warrant interventive clinical psychological therapy? If a taxonomy of CPPs is to be treatment-relevant, then not only will different CPPs imply different treatments, but the very definition of a CPP will include a criterion of being treatment-worthy.

To feel sad is a negative psychological-level state of affairs – a psychological problem. But it can also be an appropriate, constructive, natural, ‘healthy’, adaptive, or functional problem to have, as in normal grieving. What determines when this state of affairs warrants intervention? When does it become *clinical*? Is it simply a matter of degree – a dimensional criterion? Or can it be categorical – a qualitative criterion?

Table 1 lists a number of negative but overwhelmingly spontaneously-remitting psychological-level problems, and CPPs (using current DSM mental disorder labels) with similar topographies or phenomenologies. How can we tell whether a person is obsessed by food because he is being careful with his diet this week, rather than because he has an ‘eating disorder’? How can we distinguish a ‘huff’ between a husband and wife, from a relationship problem that requires intervention? One answer would be ‘Time will tell’. But what occurs differently during this time?

**Table 1:** Negative situations or reactions and their corresponding ‘psychopathological’ counterparts.‘Symptoms’ do not distinguish or define the ‘pathology’. So what does?

Psychological Problems	Clinical Psychological Problems
Grief reaction	Depression
Acute stress reactions	Post-traumatic stress disorder
Binge	Bulimic disorder
Anxious state	Anxiety disorder
Episode of substance abuse	Addiction
Misbehavior	Conduct disorder
Argument	Relationship problem
Diet	Anorexic disorder
Pain	Chronic pain syndrome
Tantrum	Impulse control disorder
Worry over health	Somatoform disorder
Fussiness	OCD

Studies of the extremely common negative situations or reactions listed in Table 1 (‘psychological problems’) show that they generally do not self-perpetuate and they tend to ease without interventive therapy. This has been found to be the case in most grief reactions [163, 164], acute stress reactions [165], the spontaneous remission of many psychological problems [166], and in all our daily experience.

What is the essential difference between a person in a depressed state, perhaps experiencing a grief reaction, and a person whose depressed state justifies, and can benefit from, interventive therapy? A person experiencing a natural, healthy grief reaction following a bereavement (a psychological-level problem, but not a CPP) can present phenomenologically quite severely. She may tick most of a symptom list. This cannot therefore define a CPP, as she may well be following a natural course toward resolution (as the majority of bereaved people do). That is, the *process* occurring – not the ‘symptoms’ or their severity – will determine whether a CPP is present and intervention is warranted. This criterion can be categorical: Either an undesired, harmful, or dysfunctional process is occurring, or it is not.

Therefore, the essence of CPPs lies in *a mechanism or process of maintenance*, which can be *discovered through functional analysis or case formulation*, and which then requires and justifies (‘clinical’) psychological-level intervention or therapy to disrupt it.

### A mechanism or process…

The overwhelming majority of people who experience the grief of loss emerge from this process without clinical intervention [167, 168]. But symptoms do not distinguish or predict who will recover within a reasonable timeframe and who will not. DSM-5 [10] merely states that a Bereavement reaction or a “normative stress reaction” may be called an Adjustment Disorder “when the magnitude of the distress…exceeds what normally would be expected” (p.289) – a dimensional, not a categorical criterion – and a diagnosis of Major Depressive Disorder may only be given after 2 weeks after the loss ([10] p.160) – a highly controversial pronouncement [169].

The most comprehensive and influential evidence-based grief theories are the Dual-Process Model of Stroebe and Schut [170] and Worden’s Task-Based Model [171]. These both describe *processes* that are natural and usually successful. Could complicated grief [172], prolonged grief [168, 173], or Major Depression be best defined and distinguished by a different *process*? Especially one that is cyclic and self-perpetuating rather than linear and progressive. This would explain why routine intervention for bereavement is not generally recommended, and “may interfere with ‘natural’ grieving processes” ([174] p.140).

### ….Of maintenance…

What is the necessary and sufficient condition that can distinguish a person simply experiencing anxiety from a person with a clinical anxiety-related problem who can benefit from interventive therapy? A comparison between an acute stress reaction and Posttraumatic Stress Disorder (PTSD) can illustrate this difference.

Around 60% of men and 50% of women will experience one or more significantly traumatic events in their lives [175, 176]. Extreme distress is common in the immediate aftermath of a traumatic event [177]. In the first weeks after a traumatic event most people experience recurring distress in response to reminders, and re-live the event in memories, dreams, and flashbacks [178, 179].

Acute stress reactions are unpleasant, and so they are a ‘psychological problem’. However, they generally fade over time [180] and most people will recover spontaneously with some support [177]. A majority of people who experience a traumatic event do not develop PTSD [181]. The lifetime prevalence of PTSD is approximately 8% 176].

Because DSM diagnosis is symptom-profile-based, and many people experience severe symptoms in the immediate aftermath of trauma, DSM has defined an interim disorder – Acute Stress Disorder (ASD). However, around the same proportion of trauma survivors with or without ASD symptoms – with other symptoms or with sub-clinical symptoms – can go on to develop PTSD [182]. Also, trauma can lead to other classes of problem, especially depression [183]. So the experience of a traumatic event and the immediate presence of ASD or PTSD-like symptoms are poor predictors of PTSD [165]. Better predictors as to whether initial learned alarms become a persistent problem and “snowball” ([180] p.15] into full-blown PTSD include accessibility of social support, and the trauma survivor’s coping style [184]. A key feature of the latter is whether it is predominantly an avoidant coping style [185–187], whether this be cognitive or behavioural avoidance [188, 189].

It is no coincidence that two of the most recommended treatment elements for PTSD are prolonged in vivo exposure therapy and imaginal exposure to flashbacks [190]. With regard to in vivo exposure, Wirtz and Harrell [191] found that either spontaneous or planned exposure to triggers associated with a trauma soon after the event reduced the likelihood of experiencing persisting distress. Such exposure seems to be how distress dissipates for the majority of trauma survivors [180]. Similarly, cognitive avoidance is undesirable when cognitive confrontation is necessary, as with obsessive or PTSD flashback problems [192].

Such a summation of this research shows that the development of PTSD from an acute stress reaction is a function of *maintenance processes* that can occur in the aftermath of a trauma. If, on the other hand, treatment is directed at *symptoms*, then this can interfere with adaptive processes. Critical Incident Stress Debriefing (CISD) and other such proactive interventive treatments administered early in an acute stress reaction have been found to be ineffective, or even counterproductive, in the prevention of PTSD [177, 193–197], just as routine intervention after a bereavement is contraindicated [174].

**Fig. 1:**
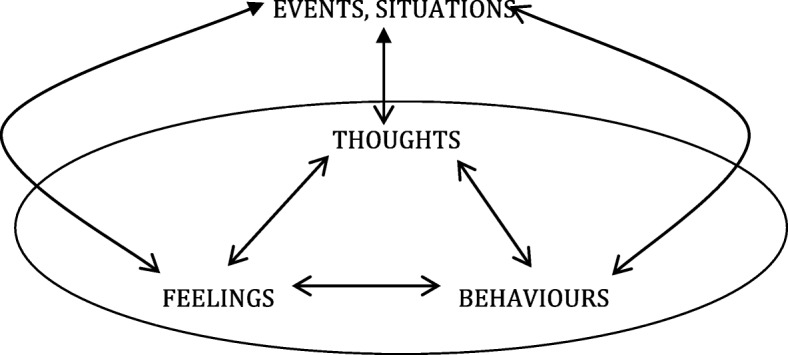
The Generic CBT Model, illustrating the essentialism of cyclic maintaining causal processes^a^. ^a^The open arrows represent normal material sequential causal pathways. The filled arrows represent A *effectively* altering B. This, and specific and general examples of each causal arrow, and the involvement of drug therapies, is fully described in [219].

This critical maintenance criterion for CPPs holds that psychological problems such as sadness or anxiety (problematic emotions), preoccupations or obsessions (problematic cognitions), or classroom disruptive behaviour or frequent handwashing (problematic behaviours) (see Fig. 1) will, being aversive, tend to resolve, diminish, habituate, or extinguish if not *maintained*. This process of maintenance, if it occurs at a psychological level, and so is amenable to psychological-level intervention, is then *what ‘causes’ and defines a CPP*. “Self-perpetuating vicious circles” have been found to explain the persistence of “symptoms” not only in grief and bereavement and in PTSD, but also in anxiety states, panic syndromes, obsessive problems, and depression [163].

Therefore, the essence of a CPP lies in some form of psychological-level maintenance process. Inasmuch as a treatment needs to address the ‘true cause’ of a CPP, the relevant maintenance process should also indicate appropriate therapeutic intervention. (See Appendix A concerning confusion over addressing the ‘true cause’, historical etiology, and underlying mechanism etiology).

### ….Discovered through functional analysis or case formulation

While many medical diagnoses point to underlying biochemical or neurological mechanisms, few psychopathological ones do [23–25]. Neither do they indicate particular psychological-level mechanisms [34, 35]. Hence, clinical psychologists rarely find such diagnoses useful.

Instead, clinical psychologists will develop a case formulation through a functional analysis of presenting problematic behaviours, cognitions, emotions, and situations or stimuli [139, 198]. The case formulation or conceptualisation “will explain the origins of the problem, account for the maintenance of the current problem, and make predictions about prognosis, [and] prescribe treatment options” ([199] pp. 89–90).

Clinical psychologists, in practice, do very little diagnosing, but much case formulation [51, 52, 200]. According to Persons [201], the purpose of such a formulation is always to direct treatment. “The case formulation links the patient’s data on the one hand with the treatment plan on the other” ([199] p. 89). Whereas in psychiatry the interview may be mainly guided by the DSM classification process, in clinical psychology case formulation is an experimental hypothesis-driven procedure in pursuit of a “clinical theory” (the problem formulation) that guides the therapy process [202].

Early forms of CBT tied to DSM’s diagnostic categories produced manualized protocols (‘a pill for an ill’). However, the case formulation approach has led much more to tailored, individualized treatments based on idiosyncratic, but evidence-based, case conceptualizations [54]. The Division of Clinical Psychology’s position statement on functional psychiatric diagnoses recommended the promotion of the use of psychological formulation rather than traditional psychiatric diagnosis ([17] p. 9).

### For example, CBT case formulation

Case formulations in clinical psychology are developed within many alternative theoretical orientations. Any new conception, and hence taxonomy, of CPPs will need to accommodate all evidence-based models of CPPs. It will need to be, in that sense, a meta-theory.

However, for present purposes, in our search for an essence to CPPs, the process of case formulation within behaviour therapy and within CBT will be examined initially.

The ‘functional analytic clinical case models’ of behaviour therapy were developed as a clear and parsimonious way to organize the variables and relationships in a functional analysis ([139] pp.31–33). These vector-graphic representations of variables and their functional relationships, involving causal arrows connecting boxed or enclosed variable labels, have proven to be an effective way to communicate behavioural case conceptualizations, and are subsequently a guide to treatment decisions [203].

This technique was adopted for individual CBT case conceptualization as well, and hence their generalized form – CBT models of psychopathology – are also often presented visually as vector diagrams [139] with cognitive, emotional, behavioural, and environmental/situational elements connected by causal arrows, which can mean “leads to”, “causes”, “allows”, “determines”, “increases”, “affects”, “enables”, “is a result of”, or “is dependent on” ([139] p., 32, [32] p., 459). Textbooks in CBT are replete with these models. Because CBT case formulations and subsequent treatments focus heavily on maintenance processes rather than historical etiological causes, almost all such models incorporate *feedback loops*, made evident by the arrows in their diagrammatic representation.

The first highly influential such evidence-based model of a CPP, incorporating a full feedback loop or ‘vicious circle’, was Clark’s panic cycle [204]. Many others have been developed since directly from the empirical research, and describing a wide range of problems, including general emotional distress ([135] p. 44), PTSD ([205] p. 321), 180 (pp. 10–11)], panic disorder ([206] p. 109), worry (p. 79), general anxiety ([207] p., 53, [208] p., 9), bulimia ([209] p. 19), anorexia (p. 21), social phobia ([210] p. 72), depression ([211] p.178, [212] p. 98)], OCD ([213] p., 127, [214] p., 80, [215]), hypochondriasis ([216] p. 261), and health anxiety ([217, 218], p., 370).

All of these models can be represented by the generic CBT model presented in Fig. 1. This is the most advanced, evidence-based candidate for “an alternative, universal theory of what maintains and exacerbates psychological distress” ([4] p. 8). The model incorporates such diverse psychological phenomena as operant conditioning, classical conditioning, cognitive mediation, expectancy effects, cognitive dissonance effects, psychoeducation, multimodal therapies, and transdiagnostic therapies [219–221].

Note that Fig. 1 is of its nature a cyclic maintenance model, not an historical etiological model. (See Appendix A.) It does not specifically include a unidirectional input causal arrow representing historical etiological factors. In CBT, historical etiology is of sufficient interest to be included in early assessment, and in a case formulation. But it rarely determines treatment, which will be primarily dependent on identification of elements in the *maintaining* causal processes [222]. Historical precipitants are less treatment-relevant, less universal, and suffer from the problems of multifinality and divergent trajectories [105].

### The network model

In parallel with the transdiagnostic movement, an alternative conceptualization of mental disorders has emerged that does not see them as latent underlying disease entities revealed by discrete symptom sets, or as labels for arbitrarily targeted sets of symptoms. McGrath [223] has observed that theoretical terms in psychology, such as “depression”, may often refer to a constellation of variables, rather than to a single latent structure.

Partly in order to explain DSM’s excessive comorbidity rates, such as between Major Depressive Disorder and Generalized Anxiety Disorder, Cramer et al. [27] have proposed a Theory of Complex Networks, in which disorders are viewed as “*networks* that consist of *symptoms* and *causal* relations between them” (p. 138). In the complex network approach “disorders are conceptualized as systems of causally connected symptoms rather than as effects of a latent disorder” ([224] p.93). The symptoms then do not *measure* a disorder, they are part of it. A disorder is thus conceptualized as “a cluster of directly related symptoms” ([27] p.140). An example could be: Chronic stress ➔ depressed mood ➔ self-reproach ➔ insomnia ➔ fatigue ➔ concentration problems ([224] p.96). Comorbidity is then a result of direct bidirectional relations between the symptoms of each disorder, for example the sleep disturbance and fatigue of MDD and the chronic worrying and difficulty concentrating of GAD ([27] p. 139).

This entirely new way to conceptualize CPPs is already heavily research-based (e.g. [225]) engenders more useful clinical research [224, 226], thoroughly explains comorbidity patterns [27, 225, 227], and is naturally compatible with a transdiagnostic process model of mental disorders [79], such as that of Nolen-Hoeksema and Watkins [105].

Also, psychopathology networks, unlike mental disorders, can extend beyond the individual. Reciprocal interactions can occur *between* people, as when a child’s sleeping problems produces parental sleep problems; both feed into behaviour problems, which then increase parental stress, and which does no good at all for the parents’ management of the child’s sleep problem ([224] p.104). A notion of CPPs derived through a complex network model can incorporate *inter*personal psychological or relationship problems.

And networks – like diagrammatic case formulations – imply intervention points. The ‘centrality’ of a symptom in a network refers to how causally connected and hence clinically relevant it is. It is recommended that one target in therapy the most central symptoms [228].

However, the network model does not as yet offer us a new conceptualization – a new “essence” of the common psychological disorders [79]. Until very recently, it has continued to view MDD and GAD as autonomous entities able to receive and send out causal effects, and has assumed the “illusion of one-way causality” between biological and behavioural levels of the system. This merely adds a dimensional layer onto a categorical disease model of CPPs. And has thus presented us with ‘fuzzy boundaries’ between diagnostic categories. Cramer et al. ([27] p.183) have asserted that the difference in the network model between “disorder” and “no disorder” is how many symptoms are “on”, or how severe they are. There are two criteria here. One would be better. And they are both dimensional criteria. *How many* symptoms need to be “on” to call a problem a CPP? And *how severe* do they have to be? No essence to mental disorders is stipulated.

This falling short (until very recently) of offering a new conceptualization of CPPs is attributable to the network approach’s assumption that mental disorders and psychopathology *arise from* or *result from* the causal interplay between psychopathological symptoms [227]. So in this model causal networks are explanatory and ubiquitous, but not yet essence-defining.

Borsboom [229] and Borsboom et al. [230] have come closer to such an essence when postulating that a comprehensive model of psychopathology could be developed if it is recognised that the networks’ biological, psychological, and societal mechanisms and causal relations can be sufficiently strong to generate a level of feedback that renders them *self-sustaining* in feedback loops that become ‘stuck in a disorder state’. Borsboom [229] describes this as a “general feature” of mental disorders. If, instead, this were to be regarded as a universal, essential, and definitive feature of psychopathology, then a true ‘paradigm shift’ would be complete.

Another major problem for clinicians with the network model to date is its complexity. Not only are there disagreements over the reliability of the general and theoretical results of network analysis methods (see [231] versus [232]), but in specific analyses, Cramer et al. ([27] p.180) admit that when etiology is conceptualized in terms of the development of a network over time, this can lead to enormous complexity, depends on numerous vulnerabilities, and will vary greatly from one individual to another. Belzung et al. [233] have pointed out that the discernment of therapeutic targets under the network model could be extraordinarily difficult. We are still missing a simplifying essence to CPPs.

What if, in “defining our disorders at the level of property clusters under-girded by dysfunctional but self-sustaining mechanisms” ([40] p.1149), we focus on the “self-sustaining mechanisms” (the systems or processes) rather than the “property clusters” (the topographic symptoms)? After all, “causal meaningful relations between symptoms…are the very stuff of which mental disorders are made” ([224] p.96).

### The need for a ‘linchpin’

So both the transdiagnostic movement and the network model have moved focus from symptom measurement and diagnosis to the case formulation of problem-maintaining processes. But no replacement conception and subsequent problem taxonomy or functional classification system has yet ensued [34].

The major drawback of psychological-level case formulation of CPPs when contrasted with the diagnosis of mental disorders is that functional analyses or case formulations can be complex, vague, and idiographic ([35] p.1153). Case formulation is relatively unreliable [51]. For example, both Persons et al. [234] and Mumma and Smith [235] have found good agreement among therapists in identifying presenting problems, but poor agreement in identifying hypothesized underlying cognitive mechanisms. Eells et al. [236] reviewed intake evaluations at an outpatient psychiatric clinic. They found 95% included descriptive information, but only 43% proposed an inferred psychological mechanism.

Superimposing a transdiagnostic dimensional model over a categorical diagnostic nosology (e.g. [237]) merely adds to this complexity. Functional analysis has remained “neither specific nor replicable” ([99] p.381). But a taxonomy of all possible problematic cognitions, and behaviours, and emotions, and stimuli or triggers would be unwieldy and arbitrary, barely explanatory, and would not define CPPs according to one or two discernible criteria.

Therefore, what is required is some form of simplifying “linchpin” [238] which could guide and standardise case formulation, aid communicability through standardisation of nomenclature, and ultimately define the presence and essence of CPPs.

## The essence: problem-maintaining circles (PMCs)

Assembling the criteria developed thus far, the items to be listed in the new taxonomy of CPPs must be: Problems, formulated at a psychological level, that warrant therapeutic intervention, and rest on an empirically-supported, theory-rich model, which parsimoniously and categorically defines processes or mechanisms that exist in the real world, are causally maintaining and hence treatment-relevant, simplify complexity, and aid in case formulation. It is also desirable that such listed CPPs can generate and organize treatment-relevant research, are codifiable, will minimize stigma, include relationship problems, and recognize and distinguish various ‘kinds’ of problem.

So, having loosed ourselves from the conceptual manacles of the mental disorder model of CPPs, we now find the requirements imposed upon our new conception to be much more exclusive and demanding. But one notion can satisfy all of the above criteria…..

### All clinical psychological problems are caused by PMCs

**Fig. 2:**
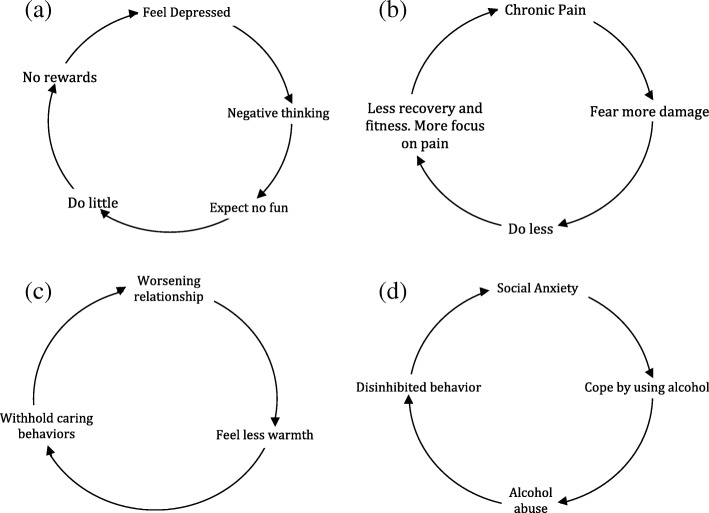
Examples of PMCs defining CPPs. Each is research-derived, generalized, explanatory, treatment-relevant, can be detected by a comprehensive psychological assessment and functional analysis, and be presented in a communicable codified case formulation. (See Appendix B for a proposed listing and coding system of such PMCs.) They exemplify PMCs within CPPs involving (**a**) depression, (**b**) a chronic pain problem, (**c**) a relationship problem, and (**d**) a 'comorbid' social anxiety problem with a causally interrelated alcohol abuse problem

It is the claim of this Proposal that the smallest, simplest ‘unit of psychological pathology’ which fulfils all of the above criteria is the functioning of a *problem-maintaining circle* (PMC) of psychological-level causal elements, several illustrations of which are presented in Fig. 2 in the form of vector diagrams. This is the simplest, most basic unit of a CPP expressed in evidence-based graphic models of psychopathology, in the generic CBT model of Fig. 1, and in the case formulations of most scientist-practitioner or practitioner-scholar clinicians. It is the ‘linch-pin’. This causally cyclic (maintaining) mechanism depicts the *essential* difference between a negative psychological-level state of affairs (a psychological problem), and a state of affairs requiring interventive treatment – a *clinical* psychological problem (CPP) (see Table 1). A CPP is then any undesired, self-maintaining, psychological-level causal cycle that involves people’s thoughts, feelings, behaviour, and situations.

Until a full PMC forms, a negative psychological-level state of affairs may be a problem, but it is not a ‘clinical’ problem warranting formal therapy. It can be expected to pass, as normal, successful coping mechanisms are employed. As soon as it becomes self-perpetuating via a PMC, such as the use of an unsuccessful coping technique (avoidance comes to mind, or rumination, or …), then intervention is justified. The identified mechanism of perpetuation or maintenance will then indicate points of intervention, and hence particular therapies.

To be upset (e.g. an acute stress reaction) is negative and psychological, so it is a ‘psychological problem’, but this does not warrant referral, formulation, and therapeutic intervention, until it persists or becomes self-maintaining (e.g. PTSD) through a process or mechanism which, when identified, can also indicate which treatment to apply. This mechanism then is the essence of, and defines, the CPP.

The simplest expression of a PMC would be a bidirectional causal relationship between two features within one element of the tripartite model of Fig. 1 (cognitions, emotions, or behaviours), as when Thought A leads to Thought B, which leads to Thought A (“I am a worthless person” ➔ “For example, I’ve failed that exam” ➔ “See, I am a worthless person”), or between two features in *different* elements of the tripartite model, as when Thought A leads to Feeling X, which leads to Thought A (“Life is awful” ➔ sadness ➔ “Life is awful”).

Since people are innately complex, many PMCs will involve several elements of Fig. 1 before a full self-perpetuating causal circle is completed, such as: Feel depressed ➔ Do little ➔ Few achievements in life ➔ Low confidence and self-esteem ➔ Do less ➔ Few pleasures in life ➔ Feel depressed.

### PMC formation, and the marginal relevance of historic etiology (See Appendix A)

If a full PMC does not form, the psychological problem (for it is still aversive, and occurring at a psychological level) will likely resolve, remit, dissolve, or be processed. For example, Bill and Mary have a screaming row. Tomorrow, Bill apologizes and life goes on. If, however, Bill wakes tomorrow still angry, ignores Mary over breakfast, she sees and resents this, there is no intimacy all week, both blame the other, her respect for him decreases, etc., then one or more PMCs have formed, and the situation qualifies as a relationship CPP which would benefit from therapeutic intervention, as it is unlikely to spontaneously remit soon.

The description of a forming PMC can start at any element of Fig. 1. For example, if someone begins to withdraw from people (a Behaviour) – whatever the reason (physical disability, poverty, illness, depressive mood, taking on a job as a lighthouse keeper, etc.) – this can lead to a socially impoverished lifestyle (Events, Situations), which can result in lowered social confidence and self-esteem, and more caution, or even catastrophizing, about people (Thoughts), which can lead to more anxiety in social contacts (Feelings), which naturally results in more withdrawal (Behaviour). Which point in this sequence is identified as the ‘start’ of the circle is somewhat arbitrary and often marginally relevant by the time therapy is sought. The initial precipitant is often no longer critical. But the behaviours, situations, thoughts, or feelings that produce a maintaining cycle are.

Generally, historical etiology of a CPP is identified in the ‘Events, Situations’ element. Common examples of such include past traumas, parenting styles, recent traumas, and stress accumulation. However, it is just as possible that ‘Feelings’ or ‘Behaviours’ or ‘Thoughts’ could “go wrong” first. Examples could include a spontaneous panic attack (a Feeling) leading to Panic Disorder, PTSD that forms from an acute stress reaction due to coping by avoidance (a Behaviour), or health anxiety that becomes problematic due to catastrophic thinking (a Thought). What “went wrong” first may be important in the *prevention* of CPPs, but is largely irrelevant once PMCs have formed. This is the reason that all recommended evidence-based therapies happen to focus on problem maintainers rather than require analysis of presumed distant historical precipitants.

### PMCs and treatment-relevance

The elements in a PMC can be specific, such as a particular thought, or general, as in an abiding attitude [219]. They can be cognitive, behavioural, emotional, or situational. Therefore each formulated PMC can implicate a number of intervention points at several levels.

For example, the ‘Feel depressed’ PMC of Fig. 2(a) may suggest interventions such as a form of cognitive therapy (to attack the “Negative thinking” element) or behavioural activation (to attack the “Do little” element) or a change in circumstances (ditto re “No rewards”). The relationship PMC of Fig. 2(c) could lead to a homework exercise of writing a list of the best features of your partner (to affect the “Feel less warmth” element), or a commitment to hug your partner three times a day (to affect “Withhold caring behaviours”).

By definition, a PMC formulation that explains the maintenance of a CPP must indicate at least one intervention point, and hence imply a known therapeutic procedure which targets this, or at least invite research into effective interventions at such a point.

### PMCs within and between ‘mental disorders’

A PMC taxonomy is clearly transdiagnostic. Some PMCs occur within the symptom network [27] of what has been traditionally regarded as a single separate distinguishable disorder, such as Depression, or Panic Disorder, or Substance Abuse. Examples of these can be found in Fig. 2(a and b), and in the numerous vector diagrams of the CBT models of psychopathology listed earlier.

However, many empirically discovered PMCs occur *between* the symptom clusters of traditionally distinguished disorders. They involve ‘bridge symptoms’, in network terms. Figure 2(d) is an example. It illustrates a ‘comorbidity’ explanation for Social Anxiety and Alcohol Abuse. Such PMCs explain DSM’s high comorbidity incidences. A comprehensive PMC taxonomy would include such recognized evidence-based PMCs. Among the most researched are the mutual relationships between anxiety and depression problems [239]. However, new ones are constantly being discovered or confirmed, such as a bidirectional association between depression and sexual dysfunction [240], between cigarette smoking and panic disorders [241], or between pain problems and PTSD [242], and precise causal links need to be uncovered in the search for therapeutic intervention points.

The voluminous and clinically valuable literature uncovering causal relationships between symptoms [27], whether they be within a cluster regarded by DSM as a single disorder, or between such disorders, currently has no place to be assembled and coordinated. Comorbidities are currently seen as theoretically disruptive. An evidence-based taxonomy of PMCs will bring together this highly therapeutically relevant information in a clinically useful way.

### Fast and slow PMCs

The PMC model of CPPs replaces the seven-or-more criteria for the existence of a mental disorder found in DSM, with one criterion: Has a psychological-level PMC formed? The nearest DSM criterion to this is duration of the problem. However, this is an inadequate criterion because the complexity of life means that some PMCs can form and warrant intervention in days, and some do not cement for months, even years. Sleep problems deserving of psychological-level intervention can form in days. If a person has a disrupted night for any reason, and then is kept awake by the anxious anticipation of another terrible night, a PMC has formed in 2–3 nights that can perpetuate or even spiral. Intervention to break this PMC may be warranted and successful even on Night 2.

On the other hand, the cognitive, affective, and behavioural changes that are expected, and possibly necessary, after the death of one’s lifelong spouse may persist for a year or longer, during which time a clinician would be loath to interfere lest the bereaved’s natural coping and recovery processes be disrupted ([174] p.140). This is due to the prolonged natural course of such grief reactions. Counseling support may be offered, but this is supportive and guiding rather than interventive of some ‘pathology’. It will understandably take a long time to be sure of, and intervene with, any PMC explaining the onset of a prolonged grieving/depression CPP.

## The need for a new taxonomy of clinical psychological problems

When the subject matter of a scientific discipline has been determined and defined, the process of study will begin descriptively, with systematic observation and listings [4]. From such activity universal principles, processes, and concepts are developed which comprise the discipline’s scientific theories. A core element of such theories has been the listings and classifications of their subject matter – the taxonomies – that each scientific enterprise has developed.

“A reliable system of classification is important for the advancement of a clinical science” ([243] p. 386) because this process within the scientific method subsequently becomes circular, such that discovered general principles, processes, and concepts then guide further observations, research, experiment, and classification ([5] p. 13). What is listed and classified – the subject matter of the discipline – is therefore critical to the advancement of the scientific enterprise, because all subsequent research is dependent upon it. We do not observe and experiment in a vacuum. What we seek and how we incorporate what we find is dependent upon our frame of reference. “Experiment is planned action in which every step is guided by theory” ([244] p. 280). “Our observational experiences are…impregnated with theories” (p. 111).

Therefore, the development of the most appropriate taxonomy of CPPs is essential for progress in clinical psychology to enable research to produce general laws and rules, to provide a common language for the reliable assessment of problems, to simplify communication, to codify problems for third-party payers, to enable relevant reliable research conclusions, to suggest problem causes, to optimize and tailor treatments, as well as to develop new ones, to inform and ease the minds of sufferers, and to organize access to services, benefits, or support groups [14, 53]. All of these functions have been performed for clinical psychology – though generally poorly – by psychiatry’s DSM, which has dominated in the administrative functions of billing and coding, insurance and service funding models, clinical communication, and research funding and approvals [101, 245].

But it has done this poorly because, though greater specificity and description of symptoms at a phenomenological level has at least given us a common language that can be applied with reasonably good interrater reliability [21, 246, 247], becoming more atheoretical to achieve greater reliability in diagnosis has sacrificed almost all claims to validity [248]. Psychiatry has tried to become tighter, stricter, and more precise at identifying made-up mental disorders.

While the British DCP has been calling for a conceptual paradigm shift [17], across the Atlantic the American Psychological Association has formally expressed concerns that DSM-5’s (re)development is not benefiting psychological treatment outcomes [11], and the president of the Association for Behavioral and Cognitive Therapies has advocated for a new cognitive-behavioural classification system for mental disorders [53]. (Note that, until a conceptual paradigm shift occurs, this proposal is still about “mental disorders” rather than CPPs.)

The current state of psychological science has been compared to that of physics 200–300 years ago, in that, despite voluminous research over the past 60 or so years, it is still plagued by theoretical disunity, which is largely a consequence of the non-standardized use of terms by researchers and clinicians in their theoretical orientation silos [249]. This disunity is reflected in an absence of clinical psychology’s own general taxonomy of CPPs.

As with clinical psychology’s own conception of CPPs, the nature of its own taxonomy of CPPs can be derived from: (1) DSM’s inadequacies; And (2) clinical psychologists’ natural taxonomic focus and categories.

With regard to (1) DSM’s taxonomic inadequacies for clinical psychology, all of the problems with DSM’s *conception* of CPPs apply to its taxonomy. Its items (mental disorders) are conceptually biologically based, not psychological-level processes. They have no psychological (or even biological) essence, and so definition and classification are only descriptive, not explanatory, and diagnosis is minimally treatment-indicative.

Due to its medical model basis, DSM diagnosis does not even tell us when to intervene medically (e.g. pharmacologically) and when to intervene at a psychological level (e.g. with a CBT), let alone whether to intervene psychologically by first addressing a person’s situation, and/or their thinking patterns, and/or their behaviour, and /or their feelings states.

Furthermore, several types of CPPs (e.g. marital problems) are not included. The stigma of a DSM diagnosis of ‘mental illness’ is barely less than that of ascribing moral weakness or sinfulness or demonic possession. And research based on DSM categories has not been able to find systematic or explanatory links between psychosocial treatments and problem types. These are the problems that a new PMC-based classification of CPPs must, and can, overcome.

Further, a new taxonomy of CPPs, based on a new conception of CPPs, must be: (a) an aid to case formulation, case conceptualization, or the functional analysis of behaviour; (b) a listing of processes or mechanisms, rather than states, conditions, or diseases; (c) a recognition of and distinction between various conceptual kinds of psychological problem, such as between ‘clinical’ versus ‘normal’, ‘psychopathological’ versus ‘psychological’, ‘organically-based’ versus ‘functional’, etc. (d) relevant to (psychological-level) treatment choice; and (e) usable in place of diagnostic groupings in research on psychotherapy outcomes.

With regard to (2) clinical psychologists’ natural taxonomic focus and categories:

## Kinds of psychological problems

As the currently proposed taxonomy is to be radically treatment-relevant, both the categories *within* the taxonomy (which treatment for which CPP?) and the borders of the taxonomy (when is treatment warranted? when is it ‘clinical’?) must be defined by this criterion. Thus far CPPs have been distinguished from other psychological problems (Table 1) by the presence or operation of treatment-warranting PMCs. Other (transitory) psychological problems, such as an anxious state or a crisis situation, may benefit from support, guidance, or information, as this can facilitate an expected adjustment/recovery process. But interventive (clinical) therapy is not indicated. The PMC model provides a useful distinction between the concepts of supportive “counselling” and interventive “therapy”.

### Psychopathological problems

But within the class of intervention-justifying, PMC-involving CPPs, surely there are different ‘kinds’ of CPPs, including ones for which psychological-level, PMC-breaking treatments are not the first-line, or best practice, or most empirically supported interventions. For example, are some problems still best regarded as ‘mental disorders’? These are likely to be problems primarily maintained by circumstances other than psychological-level PMCs, such as biochemical-level problems.

A clear candidate for a distinction between differing kinds of CPPs, that has theoretical and treatment-relevant underpinnings, lies in the distinction between predominantly organic (neurological-level) and predominantly functional (psychological-level) problems. “The current classification systems are less controversial for conditions with an identified biological aetiology such as in the fields of neuropsychology, dementias, and moderate to severe learning disability” ([17] p.2).

Because biochemistry underlies all psychological activity (all of Fig. 1) [14], it has been assumed that biological layers or levels of analysis have intrinsic causal priority [79]; that genes affect brain activity, which affects behaviour. This assumption underlies the medical model of CPPs. But we know from innumerable examples that the causal flow goes in all directions between these levels.

For example, changes in brain PET scan images of sufferers of OCD show the same effects after successful CBT treatment as after successful pharmacotherapy [250, 251]. Increases in testosterone levels can increase aggressive behaviour, but inducing aggressive behaviour can increase testosterone levels [252]. Childhood emotional maltreatment has been associated with a decrease in medial prefrontal cortex volume years later [253]. But treatment of these effects in adulthood is still best undertaken at a *psychological* level, which presumably will alter the underlying neurology.

We are dealing here simply with different *levels* of analysis. So far, brain scans have told us little new clinically and nothing new philosophically [56]. All subsequent pronouncements have thus far been “neurologisms” [254], meaninglessly adding "neuro-‘ to whatever term is being claimed to be more ‘real’ than before we imaged the concurrent neural activity. Human thought and action unfold at a number of explanatory levels and we can gather different insights at different levels of analysis ([56] p.xvii). “The view that human experience and behavior can best be explained from the predominant or even exclusive perspective of the brain” is an example of “neurocentrism” ([56] p.xix).

It is the *clinical utility* of the candidate levels of analysis and of intervention that should determine whether a problem is best regarded as a ‘psychopathological problem’ or a CPP. Therefore, are there problems that are better considered – either in therapeutic terms or in explanatory-mechanism terms – as essentially organically-based rather than entirely PMC-driven? Haslam [62] has argued that there are. He cites as examples autism and schizotypal personality. These problems may be blurred by threshold effects and multiple causal factors, but they have relatively non-arbitrary boundaries, and underlying causal mechanisms have been proposed that are not entirely psychological-level.

Further examples of problems that are difficult to account for in a purely symptom-network model [27] include schizophrenia in remission, ADHD when behaviour is controlled in a specific environment, and delusional disorder. Hood and Lovett [255] argue that the underlying disorder in these cases is latent or silent. Rubinsten and Henik [256] have added mathematical disorder, reading disorder/dyslexia, and developmental dyscalculia as almost certainly examples of the expression of underlying brain dysfunctions.

From a treatment perspective, some problems respond better to pharmacotherapy than to psychotherapy. For example, APA’s [257] practice guidelines for the treatment of patients with schizophrenia are predominantly pharmacological. Medication is considered first-line treatment for schizophrenia [258], and concurrent psychosocial interventions are “almost always offered adjunctively to pharmacotherapy” ([134] p.292). This treatment-relevance confers some clinical utility upon the DSM diagnosis of schizophrenic disorders [243].

Therefore, although CBT-based family therapy and stress management have been found to reduce the incidence of readmission with schizophrenia [134, 259–262], neither of these approaches is expected to ‘cure’ the condition, or to optimally manage the problem when used exclusively, whereas psychotherapy with most anxiety or depression problems is. CBT is not first-line therapy for schizophrenia because more than psychological-level PMCs seem to be operating.

Therefore, PMC theory seeks to distinguish problems best regarded as psychological-level, or psychopathological, or psychiatric, or neurological, or sociological, and so on, and promotes concept definition and an appropriate problem taxonomy at *each* relevant level.

Another consequence is that the common language of ‘mental health’ services is anticipated to remain that of ‘mental disorders’. This conception has dominated for a long time, and is arguably the most appropriate for a substantial portion of people’s problems. PMC theory and its conception of CPPs is likely to remain an *additional*, concordant, and complementary body of concepts and therapy-relevant taxonomy.

### Type I, II, and III psychological problems

Table 2 distinguishes three types or kinds of psychological problem within the PMC model. Type I psychological problems are aversive, undesired, negative, or dysfunctional psychological-level states of affairs that are very common, expected, normal, transitional, ‘non-clinical’, probably adaptive, have not formed PMCs, and may benefit from counseling-level support. Type II psychological problems are CPPs in which one or more PMCs are operating. They are unlikely to remit spontaneously, or transition easily, and clinical intervention is warranted and likely to be effective. Type III psychological problems are those negative psychological-level states of affairs in which the breaking of psychological-level PMCs is unlikely to be sufficient to achieve normality, and neurophysiological-level mechanisms are likely to be the primary maintainers to the problem. They may be regarded as essentially ‘psychopathological’.

**Table 2:** ‘Kinds’ of psychological problems

TYPE I General Psychological Problems	TYPE II Clinical Psychological Problems (CPPs)	TYPE III Psychopathological Problems (Mental Disorders)
Normal ‘linear’ psychological reactions.	Persistent undesired psychological reactions.	Diagnosable psychopathologic conditions.
No PMCs identified (yet?).	PMCs have formed.	Breaking psychological PMCs may not be enough. Biological PMCs may also be operating.
Refer for counseling?	Refer for clinical psychological therapy.	Refer for psychiatric (and psychological?) therapy.
Grief reactions ▪ Acute stress disorder ▪ Anxiety states ▪ Life event stress ▪ Life transitions ▪ Adjustment disorders ▪ Personality difficulty ▪ Burnout	▪ Anxiety disorders (Panic disorder, Specific phobias, …) ▪ Depression ▪ Feeding or eating disorders ▪ Obsessive-Compulsive or Related Disorders ▪ Sexual problems ▪ Posttraumatic Stress Disorders ▪ Relationship problems ▪ Substance abuse/dependence ▪ Gambling disorder ▪ Sleep problems ▪ Personality disorders ▪ Prolonged Grief Disorder	▪ Schizophrenia ▪ Schizoaffective disorder ▪ Bipolar affective disorder ▪ Dementia ▪ Delusional disorder ▪ Autism disorder ▪ Seasonal affective disorder ▪ Post-viral depression ▪ ‘Third-day blues’ ▪ Sleep-wake disorders

It must be noted that these two criteria - likely mechanism and first-line therapy - are not always consistent with each other. There are some Type III conditions for which the current best practice treatments are psychotherapies, simply due to the lack of advancement in the biochemical or neuropsychological understanding and control of the problem. Examples.

would include DSM’s Premenstrual Dysphoric Disorder, which, though assumed to have an organic basis, is better ameliorated by CBT [263–265] than by pharmacotherapy [266, 267]. Menopausal symptoms have the same standing [268, 269], as does Chronic Fatigue Syndrome [270–272], and the research and treatment guidelines are not consistent with regard to ADHD [273–275].

It is quite possible that problems will move between Types II and III classifications as psychological-level or neural-level underlying mechanisms are discovered and new treatments are developed. Again, though, these two criteria are separable. For example, neural mechanisms underlying OCD and PTSD are being uncovered [276–278], but subsequent treatments have not ensued, and for now both problems are clearly in the Type II category on the level-of-treatment criterion. This may be different in 30 years’ time.

## Kinds of (type II) clinical psychological problems

The DSM, being based on an atheoretical pragmatic model, both poorly defines the limits of its taxonomy (What is a mental disorder and what is not?), and arbitrarily categorizes its contents (What are the ‘kinds’ of mental disorder?). The lack of a defining essence to mental disorders means that, unlike the PMC-based distinction between mundane psychological problems (Type I) and CPPs (Type II), the DSM does not tell us when to treat.

But within its taxonomy DSM also regards all of the following as examples of the one ‘kind’ of thing: A bereavement reaction, a spider phobia, OCD, antisocial personality disorder, schizophrenia, autism, and frontal lobe syndrome. They are all equally ‘mental disorders’. This absence of theoretical discrimination spreads the stigma of mental illness widely, gives no guide to subsequent treatment, or to research direction, and results in unending disagreement as to kinds, categories, or classes of problems, meaning that each iteration, up to and including DSM-5, has announced major reorganizations of internal structure.

The ultimate goal of classification is its connection with treatment [21, 35, 279]. The proposed content of the new taxonomy of CPPs – indeed the very definition and essence of CPPs (i.e. PMCs) – has been determined primarily by the criterion of treatment relevance.

However, treatment relevance is only one element of the clinical utility of a taxonomic listing. Clinicians will use a taxonomy of CPPs for other purposes such as input to case formulation, for administrative, insurance, or legalistic purposes, to assist in communicating clinical information, the use of categories in clinical practice, and predicting future clinical management needs ([280] p.947). A taxonomy will be judged according to its manageability, practicality, and conformance with logical and intuitive conceptualization [280].

If classification within the taxonomy were to be according solely to specific treatment implications, this would result in categories such as “Problems treatable by exposure therapy” or “CPPs for which thought stopping is frequently a useful adjunct”. Such categories would be large, unwieldy, highly overlapping, and counterintuitive. Also, this would be a reversion to the medical model ‘pill for an ill’ or ‘Condition X therefore Treatment Y’ approach, rather than increasing the rate, reliability, and communicability of *case formulation* and subsequent tailored treatment programs.

The phenomena of divergent trajectories and multifinality [105] mean that several evidence-based therapies may be helpful for a particular problem situation, and that a particular therapy may be effective for several differing problems. This is entirely consistent with the success of transdiagnostic therapy programs and with PMC theory, in which each PMC has several elements – all possible intervention points – and any one element (e.g. hypervigilance) can feature in several different PMCs. The elements within a PMC can be specific (e.g. a thought) or general (e.g. an attitude). They can be cognitive, behavioural, emotional, or situational. Therefore each PMC can implicate a number of intervention points at several levels. The number of identified transdiagnostic processes implicating different transdiagnostic therapies is already very large [4]. And some modes of intervention, such as attention-based treatments, have been found to be useful for a large number of CPPs [281]. Grouping by specific intervention is not intuitive for clinicians [282, 283].

DSM has been criticized for ignoring the spontaneous consensus models of the clinical community [284]. Extensive studies of the conceptual taxonomies of clinicians have found that they do not match those of DSM or of ICD-10 in structure [282, 283, 285–289].

The ‘folk taxonomies’ of eclectic clinicians are quite consistent across theoretical orientations and professions [288, 289], and are guided by several aspects of clinical utility [280]. Usually included are mood disorders (excluding bipolar and cyclothymic), anxiety disorders, substance abuse disorders, eating disorders, externalizing childhood disorders, developmental disabilities, schizophrenia, and dependent and paranoid personality disorders [283]. Experienced clinicians group together anorexia nervosa and bulimia nervosa, as well as substance dependence with substance abuse, conduct disorder with oppositional defiant disorder, panic disorder with phobias and GAD, and mental retardation with autism [285]. Psychiatrists have been found to prefer fewer categories, and with flexible (ICD-11-type) guidance rather than strict criteria-based diagnoses [287]. The same preference for (ICD-11-type) flexible guidelines over (DSM-5-like) strict criteria has been found among psychologists [290]. Such classifications are generally pragmatic, and based on ‘presenting problem’.

When PMCs are grouped under presenting problems such as ‘Depression’ or ‘Anger problems’ [219], then homework sheets covering possibly relevant PMCs can be easily selected and tailored. “Some nomothetic conceptual overlay is necessary as an initial guide to the more in-depth process of individual functional analysis because it defines the domain of interest” ([99] p.380). Such categories are not formal diagnoses. Denman [291] has pointed out that case formulations will inevitably have ‘diagnostic elements’ in them, but not in a mental disorder/DSM sense. For example, “the problem is ‘marital in nature’” is a form of diagnosis and a useful label when formulating a person’s problems to determine treatment [52], so ‘marital’ or ‘relationship’ categories of PMCs would be clinically useful, but not as de facto DSM diagnoses.

The HiTOP consortium [49] has revealed a transdiagnostic, empirically-derived, hierarchical dimensional model of classification of mental disorders. Its broader dimensions (‘spectra’, such as an internalizing dimension) correspond in PMC theory to PMCs that operate among many, or even most, of our clientele. For example: ‘Develop a CPP ➔ Feel out of control ➔ Lowered self-efficacy feelings ➔ CPP is perpetuated.’ ([219] p.30.) Such broad, generic, near-universal PMCs deserve a category to themselves, as do the broader HiTOP dimensions. (This category is labelled “PMCs 11.y.z Any Psychological Problem” in Table 3 in Appendix B.)

Therefore, it is proposed that PMCs be grouped in the new CPP taxonomy for clinical utility under generally-accepted well-understood presenting problem headings such as ‘Anxiety problems’, ‘Depression’, ‘Relationship problems’, ‘Eating/Weight Problems’, and ‘Substance abuse/dependence’.

## A proposed classification and coding system

A nascent micro classification system of PMCs is already in clinical use and is taught in several postgraduate clinical programs [220, 221]. However, this listing is ad hoc, idiosyncratic, and unlikely in such an embryonic state to provide codings that would achieve broad acceptance. An effort by teams of clinicians and researchers corresponding to some meaningful fraction of the effort devoted to the development of the DSMs, is required to construct a systematic, exhaustive, and evidence-based taxonomy of CPPs in the form of distinct, identifiable PMCs. “One can only speculate how fruitful psychological research would prove to be were decades of the financial and head-space resources devoted to biological research…available to psychology” ([292] p.738).

However, in the interim, a proposed classification and coding system is presented in Appendix B. It proposes three-level numerical coding of the format “PMC x.y.z” in which ‘x’ codes represent *presenting problems* (e.g. depression), including Type III psychological problems (e.g. schizophrenia), and ‘Interactive clinical psychological problems’, currently labelled as ‘comorbid conditions’ (e.g. chronic pain & anxiety). Then ‘y’ codes represent the major classes of problematic underlying maintaining mechanisms commonly identified in several presenting problems (e.g. hypervigilance). And ‘z’ codes represent specific evidence-based mechanisms which imply specific treatment approaches (e.g. behavioural avoidance).

For example, the PMCs illustrated in Fig. 2 would code as: (a) PMC 2.2.3 (A depression problem maintained by negative thinking, which may benefit from Cognitive Therapy.) (b) PMC 6.1.3 (A persistent pain problem maintained by behavioural avoidance.) (c) PMC 3.5.3 (A relationship problem reinforced by ongoing mutual dislike/disrespect.) and (d) PMC 13.6.1 (An interactive problem in which coping by using alcohol maintains a tendency to social anxiety.)

Such a framework can systematically summarize and stimulate the currently disparate streams of research on assessment, case formulation, and therapeutic intervention in clinical psychology. For example, instead of assessing the efficacy of a particular Cognitive Therapy package in helping a diagnostically uniform (but psychologically heterogeneous) sample of sad people, subjects could be selected based upon identification, through an assessment and case formulation, of the presence or functioning of PMC 2.2.3 above. Such would be infinitely more logical, and just as methodologically sound in a randomized controlled trial.

## The PMC model is a metatheory

CBT’s substantial evidence base, and its inherent emphasis on here-and-now case formulations and therapeutic disruption of problem maintainers, make it a natural fit for the PMC model of CPPs. For example, the dimensional models being developed by the HiTOP consortium are particularly compatible with CBT approaches [120]. They are both evidence-based, empirically inclined, and transdiagnostic, and HiTOP’s dimensions are very useful in CBT formulations [159].

However PMC theory is not specific to a CBT view of the world. The Complex Network Model of CPPs, which PMC theory has grown from, “does not involve the acceptance of any particular theory about psychopathology” ([224] p.96). HiTOP’s models are “theoretically agnostic” [120]. Theorists and practitioners from a range of orientations have identified and targeted ‘vicious circles’ or ‘vicious cycles’ as a core feature of their conception of CPPs. Only the nature and level of causal elements within their formulated PMCs vary.

For example, the movement toward integration of models of psychotherapy [293, 294], has frequently pointed to cyclic processes between internal (psychodynamic) states and external (cognitive-behavioral) events [295]. Reciprocal, cyclic, self-perpetuating processes have a “pervasive role” in psychoanalytic, cognitive-behavioral, systemic, and experiential models of psychopathology [296].

Within the psychodynamic model the type of problem or disorder can be gleaned by content, but the *presence* of a disorder can be determined by “cyclic psychodynamics” [297–299]. Narcissistic personality disorder, for example, can be understood purely intrapsychically and/or as an environmentally-maintained disposition in which “what makes them continue to feel bad is how they go about trying to feel better” ([295] p.52). Short-term existential interventions have been explained as ways to break vicious circles of emotion [300].

Family systems theorists have long emphasised cyclical formulations with psychodynamic elements [301]. Indeed they have promoted this focus on ‘circular causal loops’ in problem formulations – as opposed to linear psychodynamic cause and effect explanations – as a ‘new epistemology’ [302].

Nor is defining the essence of a clinical problem in terms of vicious circles or PMCs particular to clinical psychology. It has been proposed or enacted within forensic psychology [303, 304], organizational psychology [305], and crossculturally [306]. Much of medicine describes vicious circles of organic pathology (fever, organ failure, etc.) treated by intervening in biochemical PMCs. For example, many neurodegenerative diseases are now understood in terms of “cascades” of “proteinopathies” within or affecting neurons [307].

Therefore, theorists and researchers from many theoretical orientations are able to contribute, and to utilize, the new taxonomy, only providing that the focus is on problem maintenance, and the relevant PMCs and implied treatments are evidence-based.

## Advantages of a PMC taxonomy for clinical psychologists

A PMC taxonomy overcomes almost all of the problems clinical psychologists have with DSM. Its only comparative drawback is its relative complexity. But we have seen that, for clinical psychologists, DSM’s simplicity has been gained at an unacceptable cost to its validity and clinical utility. Life is complex. People are complex. And DSM has *over*simplified them.

Unlike DSM, a PMC taxonomy does not claim that the items it lists are inherently negative. It simply lists discovered cyclic causal sequences. Whether they are viewed as desirable or undesirable is a value judgement. There are as many ‘virtuous circles’ as there are ‘vicious circles’. DSM’s controversial conceptual overreach in asserting that, for example, homosexuality or Female Orgasmic Disorder are inherently negative (because they are “mental disorders”) is thus avoided.

Psychologists do not simply attempt to alter states or reduce ‘symptoms’. They do not merely work to cheer up a sad person or to relax an anxious person. The PMC taxonomy lists problematic ongoing underlying *mechanisms* or *processes* that must be addressed. DSM’s problems with multifinality and divergent trajectories are also thus overcome.

A PMC taxonomy defines and lists *clinical* psychological problems as perpetuating cyclic processes. This simple intuitive concept at once confers an *essence* to CPPs, that is *parsimonious* (one criterion, not seven or eight), *categorical* not dimensional, and has intrinsic *clinical utility* and treatment-relevance.

CPPs, as defined within the PMC model, qualify as essential entities on the eight criteria of such proposed by Haslam and Ernst [8]. The identification of a depression PMC is *informative*. It tells us much about the person involved. Anxiety PMCs are *historically invariant*. We know of phobic PMCs from Roman times. Such problems are *discrete*. There are definable boundaries. They are *uniform*. People trapped in eating problem PMCs have many similarities. They are, by definition, *immutable*. They rarely spontaneously remit. There is one *necessary feature* (a full causal PMC). They are *inherent*; there is a sameness under the symptoms. And they are *natural*, and not simply an artificial product of people’s efforts to classify made-up mental disorders.

A PMC taxonomy is *theory-based*, be this PMC theory or evidence-based clinical theory. But, given the cyclic nature of scientific research and theory construction, the taxonomy can improve the focus and organisation of research efforts, and the reporting and assemblage of research results.

The proposed PMC coding system can *communicate* more psychologically-relevant and treatment-relevant information than can DSM’s. “Bill Bloggs has been experiencing PMCs 2.3.1 and 2.4.1 for approximately 6 months” is much more psychologically informative – it tells us much more about what has gone wrong and what to do about it – than “Bill Bloggs has been suffering a Major Depressive Disorder for approximately 6 months”.

Communicability will be enhanced not only among clinical psychologists. The medical model and PMC theory are compatible and complementary. Already, clinical psychologists accept referrals of diagnosed mental disorders, understand what is meant, do their own functional analyses and case formulations, and often feed these back to the psychiatric or physician referrer, who understands them perfectly well, and may even appreciate a PMC code summary of this formulation.

A taxonomy of PMCs can increase the reliability of *case formulation* as well as its communicability through standardization of nomenclature. A PMC-based model is also very comprehensible and communicable to clients. Quite complex graphically presented individual case formulations can be simplified, standardized, and described by separating out the individual PMCs involved.

A person who is offered a PMC-based case formulation of their CPP with its explained mechanisms, treatment implications, openings for self-help, and overlap with normality is likely to find this more comprehensible, more optimistic, more empowering, and less *stigmatizing* than being conferred with a diagnosis of a psychopathology or mental disorder.

Within the model different ‘kinds’ of psychological problem are recognised and operationally defined (see Table 2). New useful meaning is then conferred on terms such as “counselling”, “intervention”, “therapy”, and “psychopathological”.

CPPs that do not conveniently fit the mould of mental disorders, such as relationship problems, or that cross the artificial borders of separate mental disorders, such as ‘agitated depression’ or alcohol abuse triggered by and perpetuating social anxiety, are fully recognised in a PMC taxonomy. Comorbidity is no longer a theoretical quandary.

### But does a PMC taxonomy work better?!

However, as the primary criterion for any clinical taxonomy is its *treatment-relevance* [21, 31, 99, 279], the real test of PMC theory is whether it results in more treatment success.

As research around the world has been so completely dominated by the mental disorder model, it is hard to find exceptions in which problem assessment and assessment of improvement are based on a case formulation rather than a diagnosis, let alone comparisons of the two approaches with the same population. Among the few that have been undertaken, the results are clear. This has been true for behavioural therapies based on functional analyses, and then more recently CBT based on case conceptualizations.

With behavioural interventions firstly, Carr and Durand [308] found that the treatment of disruptive behaviour needed to depend on the function it was serving (its maintainer) – whether the behaviour serves an escape function or an attention-seeking function. Durand and Crimmins [309] found that the successful treatment of self-injurious behaviour also depended upon analysis and discrimination as to whether it was maintained by an attention-getting motivation, or was escape-maintained, tangibly-maintained, or sensory-maintained. Schneider and Bryne [310] produced a significantly greater boost in observed social skills and cooperative play among children with various behaviour problems when treatment was individualized rather than standardized. In a post hoc analysis, Eifert et al. [151] discovered that their agoraphobic clients who had by chance found themselves in the treatment condition that happened to target their particular agoraphobic maintenance mechanism (fear of separation, fear of embarrassment,….) did better. Iwata et al. [311] showed that the outcome of treatment of self-injurious behaviour can depend on detection and targeting of the specific factors maintaining the behaviour, not on which diagnosis subsumes it.

Unfortunately, at about the same time that functional analysis or case formulation was being imported to CBT from behavioural analysis [312], the promotion of DSM’s diagnostic hegemony was occurring. Despite this, some comparisons of case conceptualization-based treatments – where a case formulation is “a hypothesis about transdiagnostic mechanisms that cause and maintain all of the patient’s symptoms and problems” ([313] p.455) – with diagnosis-guided treatment have been undertaken. Interest in such comparisons persisted because “flexible modular treatment is closer to how clinicians actually work than are the EST protocols that target a single disorder with an inflexible series of interventions” ([313] p.456).

For example, Jacobson et al. [314], comparing structured marital therapy with a clinically flexible version, found that couples treated with the strictly structured format had deteriorated significantly more at 6 month followup.

When Litt et al. [315] compared a coping skills-based, individualized assessment and treatment program for alcohol dependence problems with a CBT packaged program, the former resulted in higher rates of abstinence and more reports of in vivo “momentary coping responses”. In a similar vein, when the reasons for drug use are analysed so that brief coping skills interventions can be matched to personality-specific motives (PMCs) for the substance abuse, treatment efficacy is improved [316].

Persons et al. [317] demonstrated that patients with “multiple comorbidities” especially benefit when empirically supported treatment selection is guided by a case formulation.

In one of the few direct comparison treatment studies, Weisz et al. [318] have found that utilizing a decision flow chart with a modular approach to therapy for children with anxiety, depression, or conduct problems (MATCH) produced significantly quicker improvement than standard treatment, which did not differ significantly from usual care [319].

Also, internet-delivered guided self-help treatment of depression that is individually-tailored has been found more effective with more depressed subjects, and with higher ‘comorbidity’ subjects than non-tailored standardized treatment or an active (online discussion) control group [320].

Fairburn et al.’s [321] transdiagnostic theory of the maintenance of all eating disorders claims that ‘bulimia nervosa’, anorexia nervosa’, and ‘atypical eating disorders’ share similar maintaining pathological processes (or PMCs, see Appendix B). So, when they added four more maintaining mechanisms to their model (e.g. concerning mood intolerance, or core low self-esteem) they were proposing four more evidence-based PMCs for the eating problems category of our new taxonomy. It is unsurprising then when Ghaderi [322] found that individualized CBT guided by functional analyses produces better results with ‘bulimia nervosa’ clients than standardized focused CBT, including in terms of percentage of nonresponders.

So, when case formulations are used to guide treatment rather than clumsy, theory-bereft diagnoses, indications to date are that the subsequent tailored treatments improve our effectiveness. The next step appears to be to improve the reliability, standardization, and validity of case formulation and treatment selection by basing therapeutic trials and outcome studies on PMC-defined rather than DSM-diagnosed experimental groups.

## Conclusions and implications

The Division of Clinical Psychology of the British Psychological Society is one of many bodies that now “believes there is a clear rationale and need for a paradigm shift in relation to functional psychiatric diagnoses” ([17] p.5). However, to date, no universal, radical, cohesive alternative to DSM or ICD for psychologists has arisen.

It is proposed that the next logical step in the scientific development of the discipline of clinical psychology is the formation and dissemination of a taxonomy of CPPs based on its own conceptual scheme and operating primarily at a psychological level. The independent conceptual, research, and clinical development of the profession has advanced sufficiently for this critical step. In fact, it is overdue; seemingly delayed by the hegemony of another profession’s corresponding taxonomy – psychiatry’s nosology of mental disorders. Benefits to the interactive process of theory development, empirical research, and clinical practice (itself a ‘virtuous circle’) will ensue.

The proposed taxonomy comprises a listing of psychological-level problem-maintaining circles (PMCs) that distinguish and explain the critical difference between transient, linear, negative psychological (cognitive, emotional, behavioural, situational) events, states, reactions, or experiences (Type I psychological problems), self-perpetuating psychological-level causal loops (CPPs) that warrant, and can be affected by, psychological-level therapeutic intervention (Type II), and problems that can persist even when psychological-level maintainers are nullified (Type III ‘psychopathological problems’).

The development of the various iterations of the DSM has involved the assignment and coordination of vast numbers of contributors, panels, committees, and task forces. For example, three of the five volumes of the *DSM-IV Sourcebook* [323] alone commissioned 150 reviews of the literature on psychological disorders. However, these reviews were based on research that was conducted largely within the DSM framework, so much circular reasoning occurred, and alternative approaches or conceptualizations were not considered [31]. Further, DSM’s broadly atheoretical approach has made it very difficult, some say impossible, for it to select, interpret, condense, confirm, and incorporate much of the research its panels review. It is a very leaky virtuous circle.

To illustrate the problem at a conceptual level, there is no evidence that the psychopathological condition/mental disorder/mental illness called “Social Anxiety Disorder (Social Phobia)” (DSM300.23) actually exists. There are no reliable or validated biological markers or criteria or measures outside clinical psychological judgement that can detect this ‘illness’. It is defined by its effects, which are attributed to its existence [52]. It is an assumption – a convention – constructed for pragmatic, sociological, or political reasons, just as the disease model of alcohol problems and the chemical imbalance theory of depression were. They were developed in an attempt to reduce stigma or encourage people to take their antidepressants. But no evidence supportive of their veracity has emerged since. In fact, some highly deleterious effects on self-esteem, self-efficacy, confidence in treatment, and expectation of recovery have ensued [324]. (Ironically, a diagnosis can thus become a self-fulfilling prophecy, and *cause* negative feelings, thoughts, or behaviour via vicious circles, such as through instilling shame or hopelessness ([14] p.69).)

On the other hand, bountiful supportive evidence has emerged for the real-world existence and operation of the psychological-level PMC: Excessive, problematic social anxiety ➔ Overly negative evaluation of one’s social performance ➔ Unreasonable persistent regrets over social contacts ➔ Excessive, problematic social anxiety. There is psychological-level evidence for the existence and operation of every one of these causal links. In fact such evidence led to the development of this model. It is an *evidence-based* model of a CPP, which in turn directly implies therapy choice. It is *treatment-relevant*, not by way of indicating which pill, or even which monolithic 12-session therapy package, can ‘cure’ the ‘Excessive, problematic social anxiety’, but by suggesting a specific Cognitive Therapy intervention to address the distorted self-evaluation of social performance – to break the PMC at that point.

The proposed new taxonomy of CPPs is therefore theory-based, treatment-relevant, evidence-informed, and pragmatic. Much research required for its development has already been undertaken. It is now a matter of framing the results (e.g. the evidence-based models of psychopathology) in PMC terms, extracting the smallest simplest units (PMCs) and listing them under generally-useful clinical categories of presenting problem. This task deserves an effort corresponding to some meaningful fraction of that which has been devoted to DSM’s development, promotion, and dissemination.

The independence and standing of the discipline and profession may then be restored, and, more importantly, the millions of people experiencing crippling CPPs may be offered more therapeutically relevant, less stigmatising, more empowering, more evidence-based, and more comprehensible, reliable, and systematic formulations of their problems.

Many uncertainties will remain despite such a clarifying taxonomy. Disagreements will continue as to whether a particular problem is best regarded as a Type I, Type II, or Type III psychological problem. This has long been a conceptual issue (What is a ‘mental disorder’?), a research-informed therapeutic issue (e.g. counselling versus CBT versus pharmacotherapy), and a territorial/political issue among the professions. A PMC taxonomy can at least clarify the conceptual issues, and guide research to address the therapeutic issues.

## Data Availability

Not applicable.

## Appendix A

Many who promote a theory-based taxonomy or classification of clinical psychological problems (CPPs) insist that it must be based on ‘etiology’ of the problem, as this can best indicate appropriate subsequent treatment (e.g. [31]). This seems to be based on the culturally-entrenched community-wide assumption that to properly ‘cure a disease’ we must find its ‘cause’ and reverse or root this out. Anything else is just amelioration or symptomatic treatment, and will not last. The disease will remain, to recur.

But the concept of a ‘cause’ in this context is often indiscriminately cited to mean two very different entities. It can refer to a precipitant - an historical etiological factor - such as an early loss or recent trauma. Or it can refer to a current explanatory underlying mechanism or process, such as an anxiety response maintained by avoidant behaviour and catastrophic thinking.

Numerous examples can be found in the literature where the ambiguity of the term ‘etiology’ has caused conceptual, theoretical, and hence clinical confusions (e.g. [14, 31, 325]). Some discussions give onset and maintenance equal weight (e.g. [35]). The transdiagnostic movement has been concerned with both distal risk factors and with proximal within-person processes [105].

But only the latter of these can provide a treatment-relevant taxonomy. It is not logical or useful to consider *any* consequence of a traumatic event to be a CPP, while *no* problem that arises without an identifiable historical cause can be a CPP.

Therefore, the critical ‘causes’ or ‘etiologies’ are current maintaining underlying mechanisms. They are what will determine treatment.

This is true even within the medical model. For example, fever and weakness can be ‘caused’ by an infection (the underlying process). Aspirin will not ‘cure’ this, but an antibiotic might. We need to know the type of bacteria to determine the treatment. We do not need to know the source of the infection to treat it. This knowledge can be very useful for other reasons – to avoid re-infection, for public health reasons, or to check for susceptibilities in the patient. But for ‘cure’, the ‘cause’ we need to know about is the *underlying mechanism*, the type of bacteria.

The same is even more true in clinical psychology. This has been found empirically. None of the frontline, evidence-supported, best practice, psychological-level therapies listed in outcome meta-analyses require historical cures. Controlled studies do not support the efficacy of rebirthing techniques, hypnotic regression, classical psychoanalysis, traumatic incident reduction, psychodrama, catharsis, or abreaction [134, 326–328].

DSM has been criticized for being atheoretical with regard to the “etiology” of its listed disorders (e.g. [31]). On the other hand, the functional analytic approach of behavioural clinical psychology has been criticized for emphasising current underlying mechanisms rather than historical causes or precipitants. It was accused by psychodynamic theorists of providing only ‘symptomatic’ or surface explanation and treatment [329]. However, the symptom substitution debates of the 1970s were resolved firmly in favour of the ‘symptomatic’ treatments [330, 331], perhaps because these treatments were not applied to ‘symptoms’ at all (a medical model term) but actually to the important *current* underlying maintaining mechanisms behind CPPs.

Even when problem onset has been examined, many or most phobias, for example, do not arise from a distinct traumatic conditioning experience [332–335]. It is often unclear where they arise, but it can be clear what is maintaining them. Avoidance is a common maintainer.

Hence, case formulations in CBT, while they may include inputs such as characterological tendencies, historical experiences, attachment issues, and precipitating events, predominantly formulate explanations of the *maintenance* of a presenting problem [54, 202, 336]. Similarly, transdiagnostic treatment protocols particularly target “common maintaining factors” [117].

## Appendix B

Based on the preceding evidence and arguments, and on the clinical experience of the use of Bakker’s [219–221] tentative taxonomy, it is proposed

- That clinical psychological problem codes be prefixed with ‘PMC’ standing for Problem-Maintaining Circle.
- That three levels of numerical coding be used, in the format “PMC x.y.z”. More would be unmanageable. Less would excessively limit the amount of case formulation/functional analytic/treatment implication information conveyed.
- That the numerical ‘x’ codes represent generally recognised, intuitively acceptable presenting problem categories. An example of a start to such a listing is given in Table 3. Included are only those categories addressed in Bakker [218, 220].
- ‘y’ codes represent the major classes of problematic underlying mechanism (PMC element) that have been identified in CBT models of psychopathology and in transdiagnostic research, and that commonly occur in one or (often) more types of presenting problem. A preliminary listing is provided in Table 4.

**Table 3:** Some proposed ‘x’ codes in a ‘PMC x.y.z’ taxonomy

1.y.z	Anxiety
2.y.z	Depression
3.y.z	Relationship Problems
4.y.z	Eating/Weight Problems
5.y.z	Anger Problems
6.y.z	Chronic Pain
7.y.z	OCD
8.y.z	Substance Abuse/Dependence
9.y.z	Sexual Problems
10.y.z	Jealousy Problems
11.y.z	Any Psychological Problem
12.y.z	Type III Psychological Problems
13.y.z	Interactive Clinical Psychological Problems
14.y.z

**Table 4:** Some proposed ‘y’ codes in a ‘PMC x.y.z’ taxonomy

x.1.z	Avoidance (implying exposure therapy)
x.2.z	Erroneous Cognitions (implying psychoeducation, cognitive restructuring, thought stopping, self-talk coaching, etc.)
x.3.z	Selective Attention/Hypervigilance/Preoccupation (implying attentional control training, thought stopping, etc.)
x.4.z	Sleep Disturbance
x.5.z	Problematic Reinforcement
x.6.z	Narrowed Coping Repertoire
x.7.z	

As earlier described, the potential list of problem-maintenance mechanisms is enormous. Table 4 only lists some of the most researched, frequently recognised, or treatment-triggering ones. This abundance is the reason that ‘underlying mechanism’ or ‘treatment implication’ is not coded at ‘x’.

(e.) To describe a problem as being maintained “by avoidance”, or “due to reinforcement” is too broad to implicate specific treatment approaches. Therefore, it is proposed that ‘z’ codes represent more specific evidence-based mechanisms under each mechanism type. An example listing is provided in Table 5.
(f.) Type III psychological problems (‘psychopathological’ problems) are those problems that are not fully explained by psychological-level PMCs, and are rarely fully ‘cured’ by interventions that disrupt them. However, PMCs are frequently involved, and psychological therapies can often help management, reduce symptomatology, or avoid episodes of relapse. For some such problems, this is currently the best form of therapy available. Table 6 lists some of the conditions currently considered to be Type III (psychopathological) problems.

**Table 5:** Some proposed ‘z’ codes in a ‘PMC x.y.z’ taxonomy

x.1.1	Cognitive avoidance
x.1.2	Emotional avoidance
x.1.3	Behavioural avoidance
x.2.1	Lack of information
x.2.2	‘Irrational beliefs’
x.2.3	Negative self-talk/rumination
x.3.1	Seeing threat everywhere
x.3.2	Seeing failure everywhere
x.3.3	Seeing dislike/disrespect everywhere
x.4.1	Low energy levels
x.4.2	Poor concentration/decision making
x.4.3	Effects of nightmares
x.4.4	Mood effects
x.5.1	Attention-seeking reinforcement
x.5.2	Tangible, concrete reinforcement
x.5.3	Escape conditioning
x.5.4	A low reinforcement environment
x.6.1	Resorting to alcohol/drugs
x.6.2	Resorting to escape/denial
x.6.3	Resorting to aggression
x.6.4	Resorting to self-harm
x.6.5	Reduced social support
x.6.6	Reduced exercise/activity
x.6.7

**Table 6:** Some proposed ‘PMC 12.y.z’ codes in a ‘PMC x.y.z’ taxonomy

12.1.z	Schizophrenia
12.2.z	Bipolar Disorder
12.3.z	Autism
12.4.z	ADHD
12.5.z	Delusion Disorder
12.6.z	Chronic Fatigue Syndrome
12.7.z	Premenstrual Dysphoric Disorder
12.8.z	

‘z’ codes in PMC 12.y.z problems (Table 6) would specify various evidence-based PMCs known to occur in some cases of the disorder, which exacerbate symptomatology, and suggest psychological-level therapies that have been shown to ameliorate, if not resolve, the condition, such as family therapy with schizophrenia or Social Rhythm Therapy [337] for Bipolar Disorder.

(g.) ‘PMC 13.y.z’ codes represent PMCs which have been found to occur *between* traditionally-distinguished clinical psychological problems, suggesting that, if detected, treatment targeting PMCs within either or both problems may be indicated. Table 7 lists examples of these, along with supportive research evidence for their existence. Comorbidity is then not a diagnostic problem. It is a naturally-occurring, informative, and explainable risk, that broadens treatment options.

**Table 7:** Some proposed ‘PMC 13.y.z’ codes in a ‘PMC x.y.z’ taxonomy

13.1.z	Anxiety & Depression [239, 338–340]
13.2.z	Anxiety & Substance Abuse [239, 241, 341]
13.3.z	Depression & Substance Abuse [239]
13.4.z	Chronic pain & Anxiety [242]
13.5.z	Chronic pain & Depression [342, 343]
13.6.z	Depression & Sexual Dysfunction [240]
13.7.z	

The interactive problems listed in Table 7 do not represent separately diagnosed mental disorders in the DSM sense. They represent clusters or fields within the symptom matrix of the network model [27]. Hence the labels are chosen for their clinical utility only. For example, ‘Anxiety’ includes PTSD, Social Phobia, etc. And ‘Sleep Disorder’ could be elevated to the status of a separate comorbid presenting problem, or remain a part of the network field incorporated in ‘Depression’, ‘Anxiety’, etc.

Much is omitted, and probably critically misrepresented, in Tables 3, 4, 5, 6 and 7. Correction of these errors is exactly what will indicate consensus evidence-based advancement of the scientific framework of clinical psychology.

## Abbreviations

ADHD: Attention Deficit Hyperactivity Disorder
APA: American Psychological Association
APS: Australian Psychological Society
ASD: Acute Stress Disorder
BPS: British Psychological Society
CBT: Cognitive Behaviour Therapy
CISD: Critical Incident Stress Debriefing
CPP: Clinical Psychological Problem
CT: Cognitive therapy
DCP: Division of Clinical Psychology of the British Psychological Society
DSM: Diagnostic and Statistical Manual of the American Psychiatric Association
EST: Evidence Supported Therapy
ET: Exposure Therapy
GAD: Generalized Anxiety Disorder
ICD: International Classification of Diseases and Related Health Problems
MDD: Major Depressive Disorder
PMC: Problem-Maintaining Circle
PTSD: Posttraumatic Stress disorder
RDoC: Research Domain Criteria
UK: United Kingdom
WHO: World Health Organisation
